# Saline nasal irrigation and gargling in COVID-19: Part II. Outcomes in Omicron and risk–benefit for self-care

**DOI:** 10.3389/fpubh.2025.1462286

**Published:** 2025-08-20

**Authors:** S. Huijghebaert, C. Fabbris, A. L. Baxter, S. Parviz, U. S. Chatterjee, D. Rabago

**Affiliations:** ^1^Pharmaceutical Science, Non-Profit Research, Antwerp, Belgium; ^2^ENT Unit, Department of Surgery, Ospedali Riuniti Padova Sud, Padova, Italy; ^3^Department of Medicine DIMED, Padova University, Padova, Italy; ^4^Augusta University Department of Emergency Medicine, Augusta, GA, United States; ^5^Infectious Diseases, Geriatrics & Post Acute Care, Bethesda, MD, United States; ^6^Department of Pediatric Surgery, Park Medical Research and Welfare Society, Kolkata, West Bengal, India; ^7^Department of Family and Community Medicine, Penn State University College of Medicine, Hershey, PA, United States

**Keywords:** COVID-19, gargling, nasal irrigation, omicron, saline, viral load, self-care, common cold

## Abstract

**Background:**

The World Health Organization recommends at-home management of mild COVID-19. While our preliminary evaluation provided evidence for saline nasal irrigation (SNI) and gargling in COVID-19, an update and risk–benefit assessment for self-care in Omicron infection is warranted, from treatment and preparedness perspectives, as new SARS-CoV-2 variants continuously emerge, while symptoms overlap with those of common colds and other upper respiratory tract infections.

**Methods:**

Systematic literature searches for preclinical and clinical studies involving Omicron infection and saline, bias assessment, and review of outcomes (benefits, risks).

**Results:**

A total of 14 studies met eligibility criteria: one experimental proof-of-concept study, eight randomized clinical trials (RCTs), two quasi-experimental, two matched case–control, and one controlled study (2,389 patients, 1,101 receiving saline). Study designs were highly heterogeneous, not allowing pooling of the data. In line with the pre-clinical findings, the clinical trials showed lower viral loads or faster viral clearance with SNI use; results were consistent, if SNI was started early in the infection. Individual studies supported reduced infectivity of saliva, inflammatory mediators and C-reactive protein, and increased lymphocytes. Symptoms resolved faster if severe at baseline, in line with the findings from pre-Omicron RCTs; the ability to perform daily activities was assessed in one RCT and improved significantly. Early initiation of daily SNI/gargling before the onset of smell/taste dysfunction prevented their development. Daily SNI hygiene was also associated with less frequent development of fever and a shorter duration of fever than observed among (non-irrigating) controls. Daily SNI modestly helped to reduce household transmission; a preliminary report suggests that reliable prophylaxis can be achieved, provided daily SNI is combined with strict use of personal protective measures. Hospitalization was virtually absent. Isotonic SNI was best tolerated.

**Conclusion:**

This analysis is consistent with prior review findings: early initiation of SNI/gargling may help patients with mild COVID-19 feel better, irrespective of the variant. If clean water and irrigation materials are provided, SNI can reasonably be recommended as early self-care for COVID-19, as it is for the common cold. Larger prospective studies are required to determine optimal protocols and SNI’s potential role in respiratory pathogen pandemic preparedness.

## Highlights

- Benefits: Iso- and hypertonic nasal saline irrigation (SNI) reduces viral loads and shortens the duration of viral shedding in adults and children with Omicron infection. The effect was observed if SNI was started early and was independent of vaccination status. Duration of viral shedding outlasted symptom duration.
- Although many studies were small, isotonic SNI was found to relieve respiratory symptoms and to help resume the ability to accomplish daily activities. SNI combined with gargling prevented the development of smell/taste disorders, if started before their onset. Symptom improvement was also observed in pre-Omicron trials and overlapping URTI. SNI may lead to decreased medication use.
- Apart from the main rinse effect of SNI (diluting and removing virus), gargling with isotonic saline (60 s) may be recommended as an adjunct to SNI to decrease infectivity of saliva, while repetitive SNI may help to enhance virus-neutralizing potency against Omicron in nasal secretions.
- Risks: SNI and saline gargling appear safe when clean water and irrigation materials are used for their preparation. No adverse effects, and only limited, spontaneously resolving side effects were noted. There was no disease aggravation. Omicron infection in these studies was not generally associated with severe disease, need for escalation in respiratory care, or hospitalization.
- In studies of immune/inflammatory markers perspectives, no adverse effects were observed; rather, if SNI was started early, reduced viral shedding was observed, paralleled by an increase in lymphocytes and a decrease in CRP and IL-6.
- Prophylaxis requires a combination of strict use of personal protective measures—SNI and gargling protocols, and optimization of the same requires further studies.
- Isotonic saline was well tolerated. Hypertonic SNI was found to occasionally cause self-limited nasal itch, pain or irritation. In children, epistaxis was noted, yet rated as non-related (also seen among controls). Adding antiviral or antiseptic ingredients was associated with more adverse effects.
- No risks were observed from a self-care perspective.
- Conclusion: Overall, the results indicate SNI with gargling can be recommended safely as a daily hygiene practice in COVID-19, including the Omicron infection. Clinical results are best achieved if started early.
- A pragmatic approach to COVID-19 self-care can include SNI with gargling as soon as upper respiratory symptoms appear, similarly to self-care recommendations for common colds and other upper respiratory conditions.
- Large prospective studies are warranted to determine optimal protocol and potential respiratory pathogen pandemic preparedness.

## 1 Introduction

With the advent of milder SARS-CoV-2 variants and increasing herd immunity to COVID-19, pandemic measures have been lifted by the World Health Organization (WHO), which notes, “Most infected people will develop mild-to-moderate illness and recover without hospitalization”. The WHO now recommends “People with mild symptoms who are otherwise healthy should manage their symptoms at home” (1). The Omicron variant mostly presents with upper respiratory symptoms like a common cold (2), yet infectivity in nasal tissues and immune escape are enhanced (3–6). While typically mild, Omicron can also progress to more serious disease, inflammatory response and Long COVID, most severe COVID-19 disease starts as a mild infection (7, 8).

While the WHO guidelines for COVID-19 do not give specific recommendations except for use of antipyretics (1), many recommendations on the Internet propose various medicines to treat COVID-19 at home for fever (antipyretics, NSAIDs), nasal symptoms (decongestants, over-the-counter antihistamines, anticholinergics), cough (antitussives, cough suppressants), and throat pain (lozenges) (9–15), including for children (16). The evidence supporting these measures is limited to symptomatic relief at best (9–15). Strikingly, saline drops, saline nasal irrigation (SNI), and saline gargling, recommended for self-care of sinonasal conditions including viral common colds and sore throat in children and adults across Europe and North America, have not received attention, yet COVID-19 is a viral upper respiratory illness, and its symptoms overlap with those of the common cold and other upper respiratory tract infections (URTI) (17–19).

We are a multi-disciplinary team with pharmacological, clinical, and research-related experience with saline irrigation in COVID-19 and related respiratory conditions. In 2022, we assessed SNI and gargling in COVID-19 (20). This initial review of studies, most prior to Omicron, suggested that SNI and gargling are symptomatically helpful to patients and may limit or prevent disease. Findings supported reduction and accelerated clearance of the SARS-CoV-2 viral load, reduced symptom severity and hospitalization. While some of the reviewed studies were small or had design limitations, our analysis reported that there is substantial *in vitro* and *in vivo* evidence for COVID-19, with minimal side effects (20). SNI and gargling are reported to be safe and inexpensive for daily use and are feasible for all ages; we, therefore, proposed inclusion of SNI and gargling as an early intervention to prevent disease and to relieve symptoms of SARS-CoV-2 infection, similar to those for the common cold or other URTI (19–25). Our study reviewed literature through November 2022 (20). Variants emerged rapidly, raising the question whether the benefits of SNI and gargling observed in prior work were also reported in studies with those variants, including Omicron. The WHO has provided new guidance on response to any respiratory pathogen, such as influenza or coronaviruses (26): one of the points is to be ready to manage mild and moderate cases in the community and through home-based care.

Therefore, we reviewed studies of clinical outcomes, mechanisms of action, benefits, and risks, when SNI with or without gargling is implemented early in COVID-19. The fact that symptoms of SARS-CoV-2 infection overlap with those of the common cold and other URTIs adds urgency to this assessment. If safe and effective for reducing viral loads and relieving URTI symptoms, irrespective of the viral pathogen, SNI with gargling may have a role as an easy-to-implement, affordable, non-pharmacological intervention for treatment and also respiratory pathogen pandemic preparedness.

## 2 Methods

**Figure 1:**
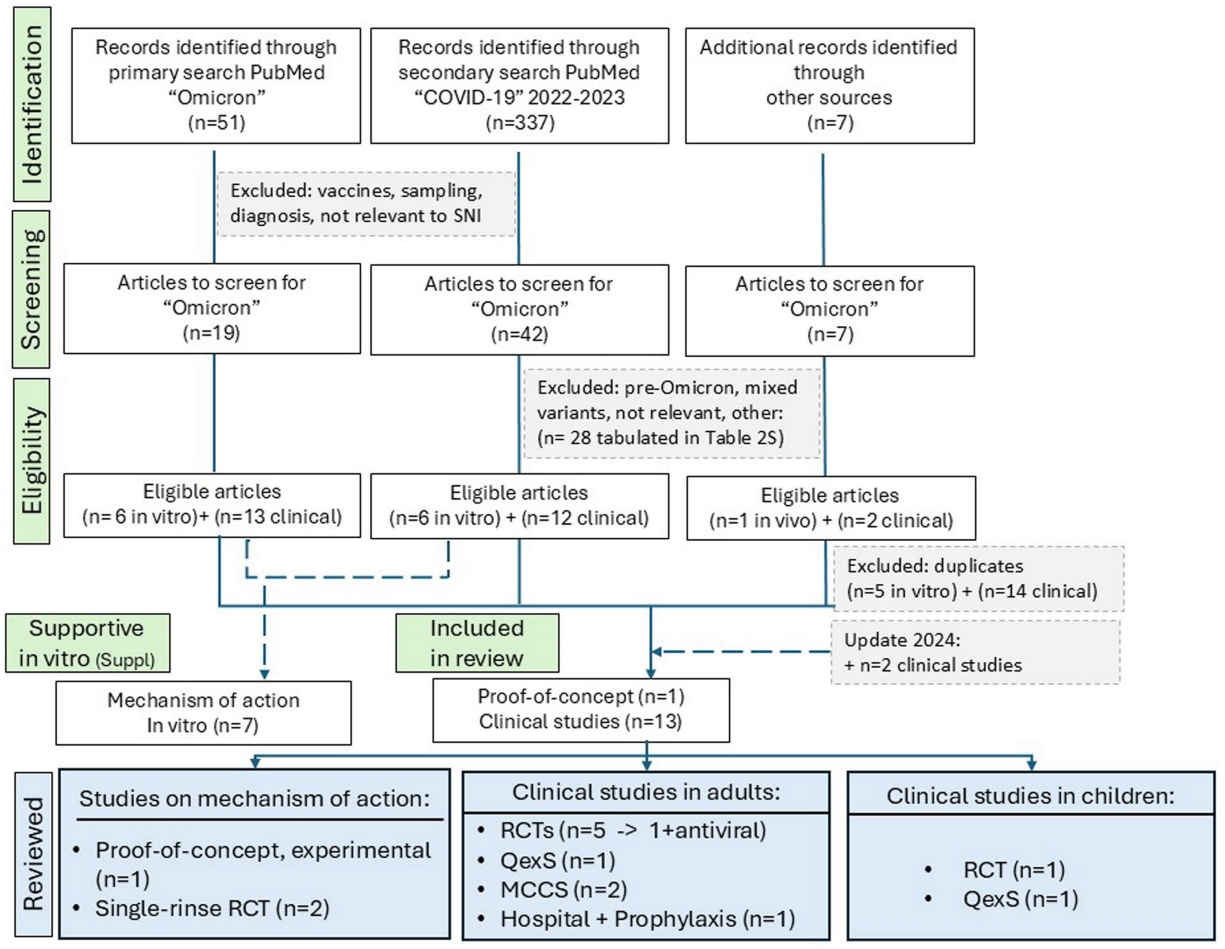
Flow diagram for the reviewed studies, identified with SNI and gargling in Omicron infection. RCTs, randomized clinical trials; QexS, (randomized) quasi-experimental studies; MCCS, Matched Case–Control Study.

We conducted a review with primary searches on PubMed using prior inclusion/exclusion criteria (20), updated for Omicron infection. We, therefore, included publications from 1/12/2022 to 29/2/2024 following the PRISMA Checklist 2020 (27). Supplementary Figure S1 identifies search terms, combined with saline, and the details of the search strategy (summarized in Figure 1), the number of studies and their main reason for exclusion. We, thereby, used the eligibility criteria implemented in our previous review [see Supplementary of ref. (20)]. In brief, the inclusion criteria allowed all types of reports and study designs, studying saline use (prophylaxis or acute treatment) as gargle and/or nasal irrigation, nasal spray or drops, or inhalation, both as intervention or placebo reference in the study, in quantitative polymerase chain reaction (qPCR)-confirmed COVID-19 patients. The exclusion criteria included studies making efficacy claims based on *in vitro* assessment, ideas, concepts, perspectives, or review material, as well as clinical studies with less than 10 patients per allocation group (unless for mechanism of action), clinical studies using saline for other purposes (such as for placebo injection in vaccines studies or diagnostic purposes); and clinical studies with comparators, not allowing ascertainment of the results due to a potential bias by a direct interaction of the substance tested with the qPCR-test [for details, see Supplementary ref. (20)]. The following other criteria were added, along with the evaluation of the material for this review: studies which included other mutations than Omicron were only included if at least 50% of patients had Omicron, the percentage of variants was specified, and randomization with regard to the different mutants was appropriate. If there was a serious concern about bias regarding a different composition of the control groups, the data were not tabulated; if only severe COVID-19 or ventilated patients were studied, the study was also excluded, as these observations are not relevant to self-care, but they are briefly discussed from a risk perspective. If a randomized controlled trial (RCT) also enrolled and randomized patients with (non-COVID-19) URTIs in parallel to COVID-19, the data from the URTI subanalysis were tabulated for the given study, grouped below the COVID-19 outcomes, under the subheading “URTI”; this to allow evaluation of risks, in view of the overlapping symptoms of COVID-19 with common cold and URTI. Studies of saline for procedural usage (dentistry, or after acute exposure to an aerosolizing infectious event) were not retained in this analysis, unless relevant to mechanisms of action. For the latter purpose, experimental proof-of-concept studies on SNI in Omicron infection were also eligible. The excluded studies with information on saline in COVID-19, that were not retained in the analysis after evaluation, and the reasons why, were listed in Supplementary Table S1. *In vitro* studies supporting the mechanistic effects of saline in Omicron infection were only considered supportive and listed in the Supplementary Table S2.

Pooling of the clinical outcome data was not possible due to the heterogeneity of study designs, patient selection (e.g., out- or inpatient, asymptomatic or symptomatic) criteria, irrigation or spray volumes, and outcome measures used. Therefore, the (pre-)clinical outcome data and the significance levels were tabulated in the supplement, and the results consolidated descriptively by (1) proof-of-concept (Supplementary Table S3); (2) mechanisms of actions from two single-rinse RCTs (Supplementary Table S4); and (3) outcomes of clinical treatment studies (Supplementary Table S5).

We evaluated patient-oriented outcomes with SNI and gargling, two pragmatic, achievable self-care measures conceptually similar to other nonpharmacological interventions in primary care or self-care for mild-to-moderate COVID-19 (28). Designs and assessments were heterogenous between the studies. Therefore, (pre)clinical results were grouped/stratified as follows (see Figure 1):

(Results part 3.1) results relevant to the mechanism of action: covering a preclinical proof-of-concept study of SNI and two clinical single-dose RCTs.

(Results part 3.2) assessment of the benefit in clinical studies: evaluation of the effects of SNI in Omicron-infected patients on viral shedding (Section 3.2.1), symptoms and daily functioning (Section 3.2.2), use with combined antiviral or antiseptic/SNI treatment (Section 3.2.3), and prophylaxis against Omicron infection (Section 3.2.4); for the interpretation of outcomes from the perspective of self-care, also pre-Omicron data on symptom relief from RCTs, reviewed in the previous review (20), were tabulated in detail and analyzed for outcomes and bias assessment (Supplementary Table S6).

(Results part 3.3) assessment of the risks, including immune/inflammatory parameters, hospitalization, progression to severe disease/ventilation (Sections 3.3.1–3.3.3). All AEs from Supplementary Tables S4–S6 were tabulated.

The parameters were grouped as follows: the effect of SNI on viral shedding was expressed as (1) change in duration of viral shedding (DVS), defined as the time from study enrolment to qPCR-negative tests (usually required on 2 subsequent days); (2) the percentage of patients testing PCR-negative; or (3) the percentage reduction of viral load on specific days after enrolment. Symptom outcomes focused on the time to symptom resolution or relief (TTSR) or the change in the ability to accomplish daily activities. Because many Omicron patients were asymptomatic, we further assessed the relevance of the findings to self-care of COVID-19 by also analyzing symptom outcomes from (overlapping) URTI, and RCTs prior to the surge of Omicron (referred to as pre-Omicron studies). The latter RCTs are listed in Supplementary Table S6 (29–36) (see Supplementary material). Other potential factors that were evaluated included the following: the combination of SNI with antiviral or antiseptics, prophylaxis, risks including the effects on immune and inflammatory markers, disease progression and hospitalization, and AEs.

Because the studies varied in sample size and, saline was the control therapy (placebo) in some studies, while SNI and gargle cannot be blinded due to its salty taste and the irrigation technique being trained at the start of a study, these limitations were taken into account for the bias assessment (Supplementary Tables S7–S9).

Although many studies were small, the study material overall included sufficient studies and patients, in order to allow for aggregate grading of evidence, by scoring the individual studies, as formerly used by Yuen et al. for nonpharmacological interventions (37). The aggregate level of evidence for the recommendations is thereby based on the quality of the studies, attributing a score 1–5 per study: score 1 for properly powered and conducted RCTs; score 2 for well-designed (also prospective comparative) CTs without randomization; score 3 for case–control studies/retrospective cohort studies; score 4 for case series and cross-sectional studies; score 5 for opinions/case reports. In addition, the preponderance of benefit over harm is evaluated to enable the attribution of an aggregate level of evidence for recommendation. The Grades A, B, C, and D correspond thereby to preponderance of benefit over harm, leading to a strong recommendation (Grade A), a strong to simple recommendation (Grade B), a simple (Grade C), or no recommendation (Grade D; more study is warranted for a reliable recommendation). The scores given by the network members per study and the resulting grade are given in the Supplementary Table S10. As per the criteria of this quality rating of studies, heterogeneity in the quality of the studies thereby leads to a lowering of the grading level; see Yuen et al., for more details (28).

Studies were rated by at least two assessors, and if disagreement, they were reconsidered iteratively until consensus was achieved. Furthermore, because saline is established as a simple effective and safe hygiene and treatment measure for other indications, the evaluation of studies included a benefit–risk perspective, as can be expected by clinicians in practice and by patients or users for self-care with this hygiene intervention. Hence, not only were RCTs taken into account, but all studies retrieved and evaluated in relation to Omicron infection, as well as the pre-Omicron outcomes formerly reviewed (20), irrespective of their study design. The main findings of the evaluation, the resulting aggregate level of evidence (grade A,B,C, or D) and the recommendations, are consolidated in a Summary Box per result section.

## 3 Results

A total of 14 studies relevant to Omicron infection were identified (Figure 1). These included an experimental proof-of-concept study (Supplementary Table S3) (37), two single-rinse randomized clinical trials (RCTs) (38, 39) relevant to the mechanism of action of saline (Supplementary Table S4) and 11 clinical studies with SNI (Supplementary Table S5). Nine of these enrolled adults (40–48), two enrolled children (49, 50), totaling 2,389 PCR-positive patients with solely (*N* = 10) (38–46, 48) or mainly (*N* = 1) Omicron infection (47). In total, 1,101 participants received saline (987 control therapy, 275 comparison therapy). *In vitro* studies relevant to the mechanisms of action of saline in Omicron infection identified during the systematic searches were summarized (Supplementary Table S2).

### 3.1 Mechanisms of action of SNI in Omicron infection

**Figure 2:**
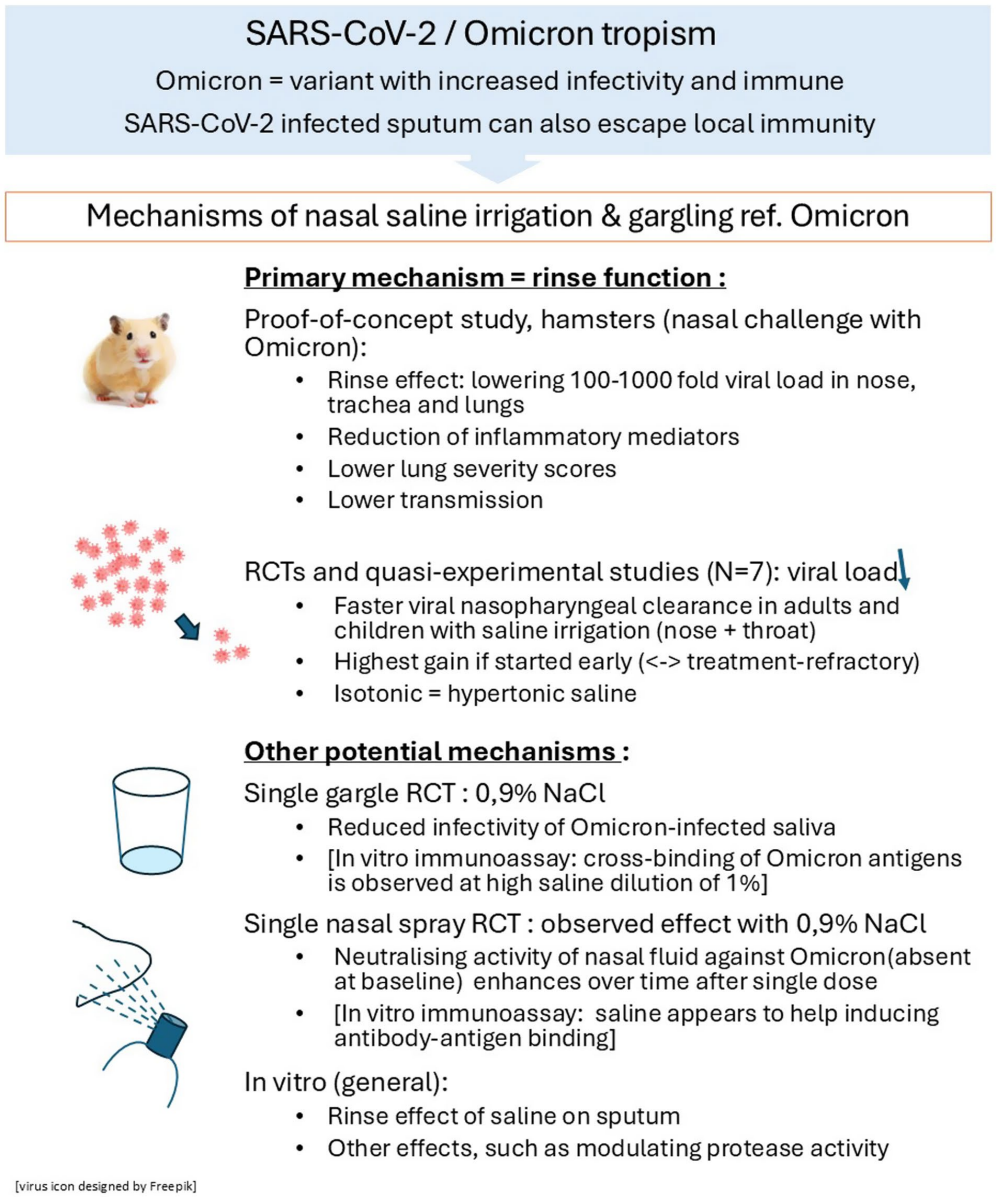
Mechanisms of action of SNI/gargling documented in Omicron infection: illustration of consolidated findings.

Figure 2 illustrates various mechanisms of action that were identified with Omicron, a SARS-CoV-2 variant which is characterized by increased infectivity and immune escape (3–6). The detailed experimental and single-rinse RCT data are available in Supplementary Tables S3, S4. Effects on viral load are reported under the benefit assessment (Section 3.2.1).

#### 3.1.1 Mechanism of SNI: proof-of-concept in the Golden Syrian hamster

In an experimental proof-of-concept study in the Golden Syrian hamster infected with Omicron BA.1, a reliable predictive model for COVID-19 in humans (37), daily use of SNI (1 mL daily) reduced by 10- to 100-fold the viral load in the nose, trachea and lungs (both RNA material and live virus; *p*-values varying between *p* < 0.02 and *p* < 0.0001, see Supplementary Table S3). SNI also efficiently attenuated inflammation and lung injury scores (*p* < 0.0001), consistent with decreased viral load. At Day 5 of SNI, interleukin-6 (IL-6, *p* < 0.005), IL-10 (*p = *0.0265), interferon-gamma (*p* = 0.0156), and tumor necrosis factor-alpha (*p* = 0.0146) were significantly reduced in nasal fluid; mRNA levels of two critical interferon-stimulated genes were increased in lung tissue, suggesting an enhanced type-one interferon response. Moreover, daily SNI reduced viral transmission by close contact, yet did not fully protect the (non-irrigated) recipient hamsters in the model (37). Ivermectin, hydroxychloroquine, and azithromycin failed to produce such effects in this model (51, 52).

#### 3.1.2 Single-rinse studies in humans

We reviewed two single-rinse RCTs with SNI serving as control. One compared single saline gargles with antiseptic mouthwash (chlorhexidine-based) on viral (RNA) load and culture-positivity of saliva (38). The other compared single-rinse virus neutralizing potency of nasal fluid after nasal saline or nasal neutralizing antibody spray (39). See Figure 2, for schematic overview of mechanisms identified.

Outcomes: Gargling for 60 s with 0.9% saline tended to reduce infectivity (TCID50) of salivary samples after 30 min (*p* = 0.0977), while the (qPCR) RNA load became as low as after the antiseptic mouthwash (38). A TCID50 of 10, or 10 PFU/mL, is the lowest infectious dose allowing reliable quantification of virus infectivity by viral culture, able to induce infection in half of the cases after intranasal challenge (53). While more participants on saline had live virus (*N* = 9) at baseline compared with the antiseptic mouthwash (*N* = 6), only one saline participant still had a load of ≥10 PFU/mL 30-min post-gargling. The investigators concluded that there was “a trend toward reduced infectivity after gargling one time with 0.9% NaCl” (38) and cautioned that “frequent use of antiseptics may exert some negative effects such as inducing potentially detrimental ecological shifts in the oral microbiota or development of antiseptic resistance in oral bacteria.”

In the RCT comparing isotonic saline placebo with a nasal neutralizing antibody spray (single application 0.2 mL/nostril) in healthy volunteers, the neutralizing antibody activity of nasal fluid was assessed in host cells challenged with ancestral, Delta, and Omicron BA.2 (39). As expected, the neutralization potency was significantly increased in the nasal fluid of the active group at both time points. Surprisingly, neutralization potency also increased in all nine nasal samples, 6 h after saline administration [Delta (*p* = 0.0939); Omicron BA.2 (*p* = 0.0625)]. The effect was not seen immediately after saline application, supporting a physiologic neutralization mechanism of saline in nasal fluid. Observations from *in vitro* immunoassays using NaCl dilutions support such effects with Omicron (Supplementary Table S2). For instance, saliva components mixed with saline were found to facilitate the induction of antibody–antigen binding (54), while another study mentions that cross-binding of the Omicron antigens takes place upon high dilution of 1% saliva (55). Whether the effects observed from these single-rinse RCTs and *in vitro* immunoassays are relevant to, and contribute to, its main mechanism in helping to clear the virus, further warrants study; for effects of repeated SNI use on immune and inflammatory markers in clinical studies, see Section 3.3.1.

### 3.2 Benefit assessment in clinical studies

#### 3.2.1 Reduction of viral shedding by SNI in clinical studies

**Figure 3:**
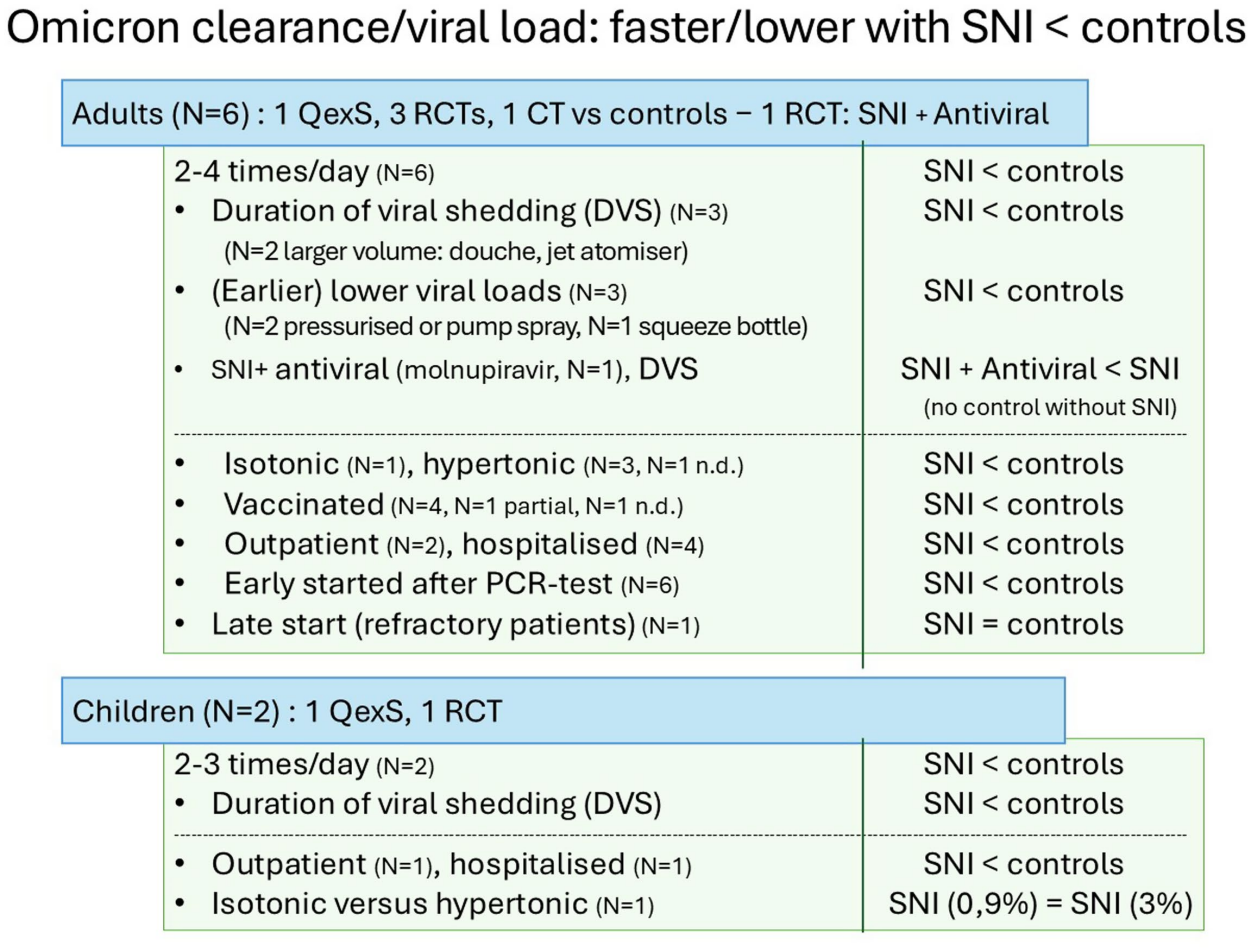
Consolidated effects of SNI on nasopharyngeal viral load in adults and children (*N* = 8). For abbreviations, see Figure 1; < means that SNI results in faster clearance or lower viral loads than controls or comparators.; = means equal efficacy.

We reviewed eight studies that assessed viral load during SNI vs. controls; six enrolled adults (40–43, 46, 47), two enrolled children (49, 50). The study results are consolidated in Figure 3. A majority of patients had been vaccinated, also including Delta (38.7%) and alpha or wild-type-infected patients (7.5%) (47). One study assessed the effect of molnupiravir add-on to SNI (46), discussed under Section 3.2.2.

Outcomes: All studies showed a consistent significant effect on viral shedding, expressed as DVS [*N* = 3 in adults (40–42) and *N* = 2 in children (49, 50)] or viral load (*N* = 3 in adults). Five used hypertonic saline, 2–4 times daily (40, 41, 43, 49, 50) and two isotonic saline, 2–3 times daily (47, 49) [one no data (42)]. For exact irrigation volumes, composition, frequencies and *p-*values, see Supplementary Table S5. DVS was significantly shortened with SNI (41, 42, 49, 50). Note that in the two quasi-experimental studies [adults (41) and children (45)], the baseline PCR threshold cycle values were very low. Low threshold cycle values indicate high viral loads and are more likely to reflect shedding of infectious live virus (56). DVS was decreased by 5 days in SNI participants compared with controls, if SNI was initiated in treatment-naive patients early in the Omicron infection. This was not the case in treatment-refractory patients who received SNI after 21 days of persisting threshold cycle values (41). Similarly, Cao et al. 2022 (42) showed a significant decrease in DVS with SNI in patients admitted to the hospital, who started early on SNI after qPCR testing. In a French RCT in patients with mild-to-moderate COVID-19, earlier reduction in SARS-CoV-2 viral load was observed with SNI vs. controls from Day 3 onwards among those with severe nasal symptoms, as well as among younger participants (18–30 years); the percentages reaching threshold cycle ≥ 42 was comparable by Days 14 and 21.

In two RCTs using SNI complemented with additional agents such as panthenol (40) or algal and herbal ingredients (43), the percentage of patients becoming qPCR-negative was significantly higher than among controls, during the initial treatment days in outpatients (40) and at Day 15 in hospitalized participants, respectively (43).

Saline use: Reduced viral load after SNI was found despite vaccination, independently of the saline strength (49), application frequency, and system used. *Aggregate grade of evidence*: B (Summary Box 1).

**Summary Box 1: Effect of saline nasal irrigation/gargling in reducing viral load**

1. **Aggregate grade of evidence:** B
2. **Benefit:** Earlier qPCR-negative conversion and modest reduction of the nasopharyngeal and salivary Omicron viral load in adults and children when started early after infection
3. **Harm**: None if the intervention is well-accepted by patient
4. **Cost:** Low
5. **Benefit-harm assessment:** Benefit outweighs harm
6. **Value judgment**: There may be a modest value to reduce viral burden, so to reduce transmission and disease severity; minimal procedural inconvenience.
7. **Recommendation level:** Option
8. **Intervention:** Optimize effect by at best combining SNI and gargling, in view of complementary mechanisms on nasopharyngeal and salivary load. Effects are already observed at nasal spray volumes (recipients with a classic or appropriate nozzle).

#### 3.2.2 Symptom improvement and daily functioning

**Figure 4:**
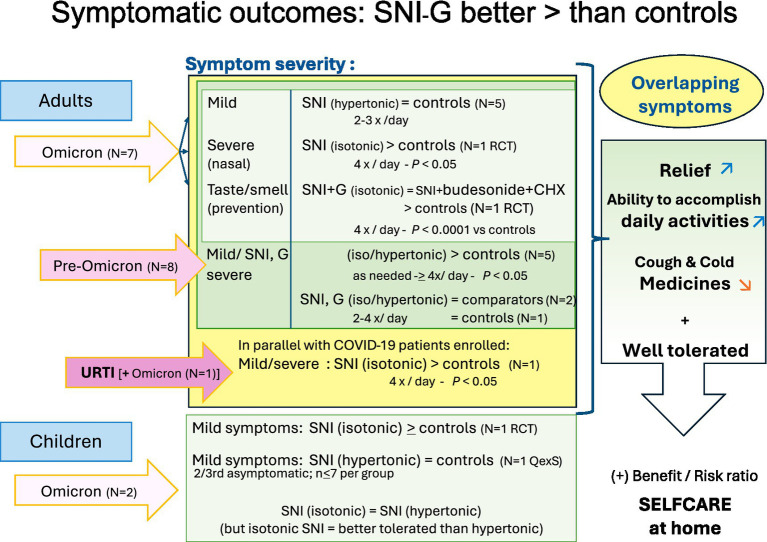
Consolidated symptomatic outcomes of SNI and gargling (G) in adults and children (*N* = 7). For abbreviations, see also Figure 1; > means SNI ± G resolves the symptoms significantly better when compared with the group of controls or comparators; = means equal efficacy.

We reviewed seven studies (Supplementary Table S5); four studies assessed symptoms (two RCTs, two quasi-experimental studies), while half to two-thirds of the Omicron-infected participants were asymptomatic; symptomatic patients mainly suffered mild illness (40, 41, 49, 50) (Figure 4). De Gabory et al. randomized patients with URTI to isotonic SNI (*n* = 177) or controls (*n* = 178), identified COVID-19 in 56% of patients (*n* = 173), yet also evaluated 146 patients with a negative qPCR diagnosis but presenting with symptoms of URTI (47). In addition, two studies assessed the effect of SNI (± gargling) on the development of symptoms: one study was a prospective case–control study assessing the effect of daily SNI on the development of fever in irrigating hospitalized patients undergoing radiotherapy for nasopharyngeal cancer (48). The other study was a three-arm double-blind, double-dummy RCT: patients received SNI plus saline spray and gargling, or SNI with budesonide nasal spray plus antiseptic mouthwash, before the onset of smell and taste disorders, as to prevent the development of olfactory and gustatory dysfunction, vs. no intervention (control group) (44): SNI plus nasal spray and mouthwash were administered one time and four times daily, respectively.

Outcomes: In the quasi-experimental studies in adults (41) and children (49), TTSR was not significantly reduced; however, these studies may have been underpowered (only seven participants symptomatic), and symptoms may have been too mild to detect an effect if one was present. Also, in a larger pediatric RCT (50), TTSR was not significantly different between the two groups, despite the significantly reduced DVS. Stuffy and runny nose decreased rapidly with SNI in the first days, while the study may have suffered from attrition bias due to selective loss of the children from the study once they were PCR-negative (50). In an RCT comparing patients receiving standard of care with vs. without nasal spray composed of seawater, xylitol, panthenol, and lactic acid (40), the trends of symptoms in both groups suggested fast resolution of symptoms in both the groups. There were, however, differences in the use of concomitant medication [antipyretics (*p* < 0.001), antitussives (*p* = 0.071), and medicinal nasal spray (*p* < 0.001)].

In the RCT by de Gabory et al., earlier recovery of the ability to accomplish daily activities (−1.6 days, *p <* 0.05) was observed in COVID-19 patients performing nasal washes of isotonic seawater four times daily for 15 days, when compared to controls (47). Symptom resolution with SNI was significantly more substantial in the subanalysis of COVID-19 patients with severe nasal congestion or rhinorrhea at start of the trial (for instance, −4.5 days for the ability to accomplish daily activities, *p* < 0.018; −4.1 days for postnasal drip, *p* = 0.037 and −3.3 days for sore throat, *p* = 0.0319). In the analysis of patients with URTIs (non-COVID-19), RCT symptom recovery was also accelerated (for instance, −8.4 days for cough/dry cough, *p* = 0.014; −5.9 days for postnasal drip, *p* = 0.04). For more data, see Supplementary Table S5.

In a case–control study in COVID-19 patients on radiotherapy, performing SNI two times daily, patients developed less frequently fever (37.7%) compared with patients on radiotherapy not performing SNI (61.5%, *p* = 0.03), non-irrigating patients undergoing other interventions (54.8%, *p* = 0.003) and the control group of nurses (98%; *p* < 0.001). SNI patients’ fever duration was also significantly shorter.

In the RCT, starting SNI and gargling before the onset of olfactory and gustatory dysfunction, 11% developed smell/taste disorders, vs. 40% among controls (*p* < 0.001), while doing equally well as budesonide nasal spray/chlorhexidine mouthwash (8.3%). If symptoms developed, their severity was significantly lower with SNI and gargling than among controls (*p* < 0.02–0.002). Former studies with SNI and corticosteroids gave less consistent results, but all started with SNI after the onset of smell/taste dysfunction (57–59).

Detailed re-analysis of eight pre-Omicron RCTs (*N* = 623; 262 saline, 104 controls, 257 comparators; Supplementary Table S7) showed that TTSR was on average longer with the earlier, more symptomatic variants of coronavirus than with Omicron. Both isotonic saline (*N* = 4) and hypertonic gargling (*N* = 1) studies reported significant reductions in TTRS and/or severity of nasal, throat and respiratory symptoms with SNI vs. controls or comparator (*N* = 5), or comparable to comparator (*N* = 2). Overall, the analysis also revealed a high variation in symptoms and TTRS among the different COVID-19 symptoms: fever and sore throat were usually short-lasting; nasal congestion and cough were longer-lasting, and taste/smell disorders were variable and potentially persisting 4 weeks or longer, also with SNI. Hence, RCTs pooling the total symptom scores (*N* = 1) (34) may fail to identify significant symptom improvement.

*Saline protocol*: Omicron studies assessed isotonic (47, 49) or mild hypertonic saline (2.3–3%) (40, 41, 43, 49, 50); others did not specify the saline strength (presumably isotonic) (42, 44–46). One study compared isotonic with hypertonic saline but found no significant differences (49); this is consistent with an RCT comparing two saline strengths (0.9% vs. 2.3%), yet also including patients infected by variants prior to Omicron (their proportions not specified per treatment groups and a large group of historical controls) (60). The role of nasal solutions containing additional ingredients, such as xylitol, is unclear. A pre-Omicron study of xylitol spray, compared to saline placebo in COVID-19, reported that saline provided significantly better relief of nasal congestion compared to xylitol (35). The application frequency of SNI may also play a role: in a pre-Omicron RCT in patients over 55 years (mostly with high BMI), comparing two different isotonic SNI regimens, symptom resolution was two times more likely with SNI two times daily (79.3% after 2 weeks) compared to SNI one time daily (42.4%; *p* = 0.003) (61). *Aggregate grade of evidence*: B (Summary Box 2).

**Summary Box 2: Effect of saline nasal/throat irrigation in reducing symptoms**

1. **Aggregate grade of evidence:** B
2. **Benefit:** Despite the effect on viral shedding, no improvement in mild omicron symptoms, yet significant faster recovery of daily activities and the spectrum of symptoms if suffering from a severe runny or stuffy nose. Early initiation of combined SNI and gargling before the onset of taste/smell dysfunction reduced the risk of developing such dysfunctions. Using daily SNI was associated with less development of fever. Prior review has also reported that symptomatic benefit was observed in studies with earlier SARS-CoV-2 variants. Symptom severity in mild Omicron infection may be too low to easily detect improvement.
3. **Harm**: None if the intervention is well-accepted by patient
4. **Cost:** Low
5. **Benefit-harm assessment:** Benefit outweighs harm
6. **Value judgment**: With minimal inconvenience, there may be a reduction in development of fever, nasal symptom burden, and smell/taste dysfunction, if SNI /gargling is implemented early. In line with observations of the common cold, SNI may reduce the need for other medications. Faster recovery of daily activities was shown in one RCT.
7. **Recommendation level:** Option
8. **Intervention:** In mild-to-moderate COVID-19, SNI can be initiated early as self-care, to reduce symptom burden, accelerate recovery, and at best even before the onset of smell and taste dysfunction to prevent progression to severe disease. Combining SNI and gargling is recommended. Both iso- and mild hypertonic solutions and various volumes and application frequencies have been used, and repetitive application daily has led to faster symptom resolution.

#### 3.2.3 Use with combined antiviral or antiseptic/SNI treatment

We assessed one RCT comparing the combination of molnupiravir with SNI as part of standard-of-care vs. SNI/standard-of-care alone (46); one retrospective matched-case controlled study, assessing rebound after rinsing with SNI plus the antiseptic polyvidone iodine for 1 week, initiated after hospital discharge for Omicron infection (45).

*Outcomes:* Antivirals are recommended in an outpatient setting in COVID-19 patients at a high risk for progressing to severe disease, to be started within 5 days of the onset of symptoms (62). In the RCT with molnupiravir (46), DVS was significantly faster with the combined regimen (median 9 days), with a median gain of 1 day compared to SNI/standard-of-care (median, 10 days; *p* = 0.0092). Early clearance to PCR-negative status on Days 5 and 7 was accelerated with the combination. Symptom relief (median TTSR) was not significantly different between the treatment groups. Molnupiravir alone (without SNI) was not assessed.

In a matched-case control study, a significantly higher proportion of cases (85.2%) had used SNI plus antiseptic in the week after hospital discharge in the group staying qPCR-negative up to Day 60, when compared with those becoming q-PCR-positive again (45.7%; *p* < 0.001) (45). The investigators recommended SNI to decrease rebound infection and transmission, but did not address whether iodine played a role (apparently added upon saline preparation), nor whether patients would have benefited from sustained SNI up to Day 60, to further reduce rebound.

*Saline use*: SNI was safely combined with an antiviral; strength and frequency were not reported and require further study. The role of adding an antiseptic to saline, such as polyvidone iodine, for SNI or gargling also needs further study: this antiseptic added to saline is associated with conflicting results compared with saline (36, 61, 63, 64), while it can also lead to reversibly changed thyroid-stimulating hormone levels over time (65). *Aggregate grade of evidence*: C (Summary Box 3).

**Summary Box 3: Effect of saline nasal/throat irrigation in combination with antivirals or antiseptics**

1. **Aggregate grade of evidence:** C
2. **Benefit:** Improvement of parameters typically predictive of worse outcome in COVID-19, provided if started early (role in refractory patients or after clinical decline unclear)
3. **Harm**: None
4. **Cost:** Low
5. **Benefit-harm assessment:** Preponderance of benefit over harm
6. **Value judgment**: while robust RCTs are lacking to document a reduction in risks (such as hospitalization) with SNI/gargling, also with earlier variants, we find no reason to withhold SNI, which is associated with improvement of clinical parameters that are predictive of outcome in COVID-19. A risk reduction in hospitalization may remain difficult to document in view of the mild prognosis of Omicron infection.
7. **Recommendation level:** Option to start early in the infection
8. **Intervention:** Vulnerable patients at risk may benefit from early SNI. Overall, SNI may be recommended early at the onset of respiratory symptoms, irrespective of testing results. Use SNI at least two times daily—up to every 4 h in case of moderate COVID-19 or pneumonia. Benefits unclear in acute respiratory distress syndrome.

#### 3.2.4 Prophylaxis against Omicron infection

We assessed the effect of prophylaxis from a published letter to the editor (42) and a prospective study in hospitalized patients treated for nasopharyngeal cancer (48). We assessed the effect on transmission in a RCT using daily SNI with seawater compared with controls (47).

*Outcomes*: Healthcare systems have started to include SNI as part of prophylactic measures (42, 66). In a letter to the editor (42), Cao et al. reported on the outcome of a multimodal prevention strategy that includes the use of SNI in a large urban COVID-19 designated hospital in Shenzhen as part of a “zero” transmission policy. They reported zero infections among medical staff even during the peak hours of the pandemic at the hospital, at which time 1,930 patients were admitted to both wings per single day; hospitalized patients also performed SNI (42, 67). Prophylactic SNI use in healthcare workers was applied in strict combination with personal protective measures, such as supervised donning and removal of personal protective equipment (PPE), immediate on-site hygiene intervention upon exposure, and isolation (+molnupiravir prophylaxis) following medium-to-high risk of hazardous exposure to a contaminated environment. The result contrasts to that of a prospective hospital study, in which the contraction rate of Omicron infection among exposed, daily tested non-irrigating healthcare workers was 100%; in the latter study, 77.6% of patients, treated with radiotherapy for nasopharyngeal carcinoma, performing SNI two times daily and weekly tested for COVID-19, contracted Omicron infection, vs. 86.7% of such patients not performing SNI as controls (*p* = 0.253 between both patient groups; *p* < 0.001 for the comparison with healthcare workers) (48). It is, however, to be recognized that the latter group may have been more exposed to COVID-19 than the patients.

In an RCT assessing isotonic seawater irrigation 4 × daily in participants with mild-to-moderate COVID-19 symptoms (47), the transmission to household contacts was not different on Days 1–21 between the active and control group, but significantly lower with SNI in the subgroup with high viral baseline loads (≥5 log 10 copies/μL) (0–23.8% vs. 0–36.4% for Days 10 and 11: *p* < 0.02). The effect was more substantial with the Delta variant (*p* = 0.04 between Days 5 and 11).

*Saline protocol*: Normal (isotonic) saline was used while applying strict PPE in the hospital (42). Effective prophylaxis with SNI has been found under similar conditions in pre-Omicron studies (20, 28). Trials that made no specific references to obligatory masking may have been less rigorous in applying PPE (47, 48), providing only limited protection. *Aggregate grade of evidence*: C (Summary Box 4).

**Summary Box 4: Effect of saline nasal washing to prevent transmission**

1. **Aggregate grade of evidence:** C
2. **Benefit:** If SNI is combined with strict protective measures, it may help protect HCWs. Limited reduction in household transmission.
3. **Harm**: None obvious
4. **Cost:** Low
5. **Benefit-harm assessment:** Preponderance of benefit over harm, use in combination with other protective measures such as appropriate mask wear, hand washing and other protective measures
6. **Value judgment**: Limited evidence available, from Omicron and pre-Omicron variants, suggests SNI has the potential to protect HCWs, and vulnerable patients in hospital and other environments at high risk of exposure, combined with other protective measures.
7. **Recommendation level:** Option for HCWs if wearing masks, and for vulnerable patients, and their siblings
8. **Intervention:** Employ as part of a hygiene strategy in healthcare and community settings. Explain the limitations and the need for other protective hygiene measures.

### 3.3 Risk assessment in clinical studies

#### 3.3.1 Immune and inflammatory markers

We assessed two quasi-experimental studies (41, 49) and one RCT combining with molnupiravir (46). To allow for interpretation, two more studies assessing such markers in severe COVID-19 (listed in Supplementary Table S1) are commented on (68, 69).

*Outcomes immune/inflammatory markers:* In COVID-19, lymphocytes are often reduced (indicating low immune response), while enhanced inflammatory markers such as C-reactive protein (CRP) and cytokine IL-6 are significant predictors of severe or Long COVID (70–72). The quasi-experimental studies revealed that, consistent with the reduction of viral load, SNI was associated with enhanced lymphocyte blood count in children and treatment-naive adult patients with Omicron infection, while this parameter remained low/unchanged in untreated COVID-19 controls (41, 49) or treatment-refractory patients (41). Similarly, CRP was significantly reduced by SNI if initiated early, while it remained static or increased in the control groups and treatment-refractory patients (41, 49). The few Omicron patients presenting with pneumonia at study entry showed improved lung injury on CT scans (41). In children (49), CRP values were only marginally improved with iso- and hypertonic SNI vs. baseline (*p >* 0.05), but CRP increased in the control group. In the study assessing molnupiravir, IL-6 and CRP normalized equally well with SNI/standard-of-care alone as if combined with molnupiravir, despite higher baseline IL-6 in the SNI group at the start of treatment in the SNI/standard-of-care group (46). It is noteworthy that the findings are consistent with those obtained in more severe disease (68, 69) (Supplementary Table S2): CRP/lymphocytes improved with isotonic SNI if early initiated in ARDS patients (68), while remained unaffected with inhalation of SNI 5% in treatment-refractory ventilated patients, albeit other parameters appeared to improve (69).

#### 3.3.2 Disease deterioration and hospitalization

For this risk evaluation, the data were collated from all RCTs reviewed (Supplementary Tables S4–S6) and complemented with data from pre-Omicron RCTs (Supplementary Table S7) for interpretation.

*Outcomes risk:* Deterioration and/or hospitalization were rare during mild-to-moderate Omicron infection, yet if recorded, were less frequent, to absent, with SNI. In a prospective study of 468 hospitalized patients on radiation therapy for nasopharyngeal cancer, none developed severe COVID-19 (48). In the RCT by de Gabory et al., rare exacerbation from mild/moderate to more severe COVID-19 was lower among SNI users than among controls: only one subject deteriorated in the third week, requiring breathing aid and oxygen therapy, yet belonged to the control group (47). Also, in a pediatric RCT, one out of 200 children in the control group was hospitalized (reason not specified) (50). In the study by Pantazopoulos et al. in hospitalized adult patients with pneumonia (43), two of 28 controls required respiratory support escalation, one dying (none in the SNI group).

*Saline Protocol*: Both iso- and hypertonic saline were used. To date, no studies have evaluated the effects of SNI on Long COVID. Overall, the lower risk for deterioration and hospitalization, also observed in our previous review (20) and in a recent non-RCT (60), the faster reduction of viral loads (Section 3.2.1), a protective effect of SNI against fever (48), and smell and taste disorders (44), and the reduction in inflammatory markers (Section 3.3.1), all suggest SNI and saline gargle as an important future area of investigation. *Aggregate grade of evidence: C* (Summary Box 5).

**Summary Box 5: Effect of saline nasal washing on ‘risk’ associated with COVID-19 (immune, inflammatory markers, hospitalization)**

1. **Aggregate grade of evidence**: C
2. **Benefit**: Improvement of parameters typical predictive of worse outcome in COVID-19, provided if started early (role in refractory patients or after clinical decline unclear)
3. **Harm**: None
4. **Cost**: Low
5. **Benefit-harm assessment:** Preponderance of benefit over harm
6. **Value judgment**: while robust RCTs are lacking to document a reduction in risks (such as hospitalization) with SNI/gargling, also with earlier variants, we find no reason to withhold SNI, which is associated with improvement of clinical parameters that are predictive of outcome in COVID-19. A risk reduction in hospitalization may remain difficult to document in view of the mild prognosis of Omicron infection.
7. **Recommendation level:** Option to start early in the infection
8. **Intervention**: Vulnerable patients at risk may benefit from early SNI. Overall, SNI may be recommended early at the onset of respiratory symptoms, irrespective of testing results. Use SNI at least two times daily—up to every 4 h in case of moderate COVID-19 or pneumonia. Benefits unclear in acute respiratory distress syndrome.

#### 3.3.3 AEs—tolerability

We assessed all included studies for AEs (Supplementary Tables S4–S6). AEs were tabulated in Table 1.

**Table 1:** Overview of AEs with SNI and gargling, as listed in the reviewed studies Supplementary Tables S4–S6.

AEs—main findings	SNI	Controls, additive or comparator	References
Isotonic (0.9% seawater) vs. controls			
Adults: ‘well tolerated’ Total AEs Nasal burning Children: AEs not-related: Nasal pain Epistaxis	(buffered SNI) 4.3% 0.3% (Physiological SNI) 0.5% 3.0%	2.8% 0% 1.5%	de Gabory 2024 (47) Lin 2023 (50)
Comparison of isotonic and hypertonic SNI			
Isotonic is better tolerated than hypertonic in children: Isotonic 0.9% NaCl Hypertonic 3% NaCl: Total AEs Mild itch Mild pain	(Isotonic) 0% (hypertonic) 25% 15% 10%	0% 0%	Liu 2023b (49)
Saline plus added ingredients			
+ Algal ingredients: Total AEs Nasal irritation	0%	+ Algae: 10.7% 10.7%	Pantazopoulos 2023 (26)
+ Xylitol, panthenol	-	+ Xylitol: 0% ‘related AEs’	Cegolon 2022 (40)
+ Antiviral molnupiravir: Total AEs Elevated ALT Rash	SNI alone: 0% 0% 0%	+ Molnupiravir: 2.6% 1.3% 3.9%	Zou (46)
Comparison with nasal corticoid			
Budesonide nasal spray Total AEs Headache Nausea-dizziness Throat irritation Nosebleed/dry nose	Isotonic saline 15.0% 5.1% 2.0% 5.1% 1.0%	Budesonide* 22.0% 12.0% 12.0% 6.7% 3.8%	Ezer 2022 (32)
Comparison with antiseptic agents			
0.5–2% PVI Nasal burning Sneezing Headache Ear pain Nasal bleeds	Isotonic saline 16.7% 8.3% 16.7% 0% 0%	0.5-2% PVI*, ƒ 28.5–92.9% 28.5–64.3% 14.3–42.9% 7.1–7.1% 7.1–14.3%	Zarabande 2021 (36)
Comparison – other			
Nasal neutralizing antibody spray: Rhinitis	Isotonic saline 0.8%	Antibody spray* 6.6%	Imsuwansri 2023 (39)

*Less AEs with saline than with comparator: for significance levels, see Supplementary Tables S4–S6. ƒ The percentages on the left and on the right right represent the mean rates of AEs observed with 0.5 and 2% PVI, respectively

Outcomes and saline use: Many studies did not report on AEs, while others referenced general safety and the lack of AEs. Overall, isotonic saline is well tolerated, with fewer AEs than nasal corticosteroids (32), mouth rinses such as polyvidone iodine (36, 65), and hypertonic saline integrating herbal or other additions (43) or comparators, such as nasal neutralizing antibody spray (39) or molnupiravir (46). While isotonic SNI was better tolerated than hypertonic SNI in children in one study (49), both saline strengths were associated with some self-limited side effects, including nasal itch, nasal discomfort; epistaxis was identified as not-SNI related and was also seen among controls (50) The findings result in a positive Benefit/Risk ratio (Figure 4). While not addressed in the RTs assessed in this review, but as pointed out in our former review (20), attention needs to be paid to prepare the saline with clean water and to use clean irrigation materials.

## 4 Discussion

**Figure 5:**
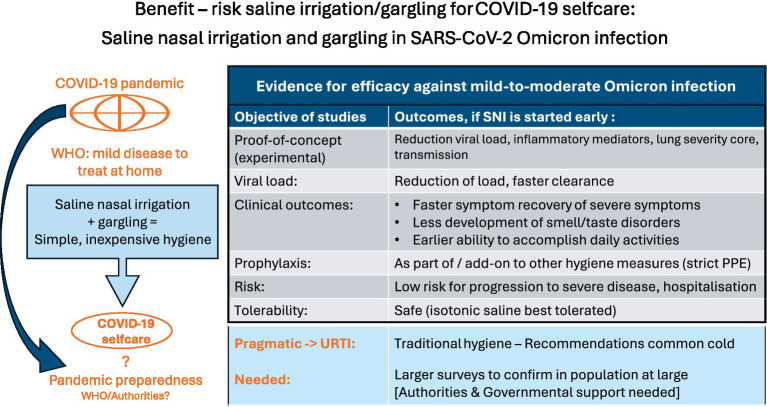
Summary slide: review the clinical findings of relevance from the perspectives of self-care and pandemic preparedness.

The key findings of this review are summarized in Figure 5 and include the proof-of-concept of SNI and the confirmation of the efficacy of SNI and gargling in reducing the SARS-CoV-2/Omicron viral load, fastening viral clearance, resolving severe symptoms and preventing development of smell and taste disorders or from mild to severe disease, if started early in the course of the infection.

The findings of this review increase our understanding of the mechanisms of SNI and its clinical effects and may help current treatment and pandemic preparedness against future variants and other future respiratory pathogens. The effects of SNI in proof-of-concept studies are relevant to humans; particularly the role of nasal rinse volumes on nasal viral loads is particularly well supported (Aggregate level of evidence B). Moreover, SNI in human SARS-CoV-2 infection may be more important than in animal models because the mucus and nasal secretions of SARS-CoV-2-infected patients have been found to be highly infectious and can escape immunity, irrespective of the symptoms or vaccination status (4, 5, 73, 74). The clinical data consistently showed that SNI reduced the Omicron oropharyngeal viral load or shortened the DVS in children and adults. The outcome received Grade B rather than Grade A, despite the consistency of the observations, because the outcomes were generally generated through a number of smaller (non-R)CTs, not only with different trial designs, but also different ways of assessments (Summary Box 1): the results were observed irrespective of saline concentration, initial viral loads, and vaccination status. The latter is relevant because vaccination does not preclude carriage of high viral loads and viral transmission, while Omicron-related hospitalization is associated with high viral loads (75–79). The proof-of-concept study (37) and single-dose RCTs (38, 39) in humans revealed plausible physiological mechanisms beyond the generally acknowledged rinse action of SNI, which is lowering of the nasopharyngeal viral loads and/or faster shedding. Although the effect on immune response parameters was not set as primary variables for saline in the studies reviewed in Section 3.1 (rather functioning as the placebo reference in RCTs), the findings together with those reviewed under sections 3.3.1 and 3.3.2 overall provide preliminary evidence that SNI may lower inflammatory mediators, lung severity scores and transmission, while enhancing viral neutralization capacity. The experimental data also support that SNI leads to less aspiration of virus toward the lungs, as lung injury scores were reduced with SNI, consistent with the significantly reduced nasal, tracheal, and lung viral loads (28). The overall clinical results with Omicron (Section 3.3) and previous variants (20, 60, 61, 68) support this concept (Summary Box 5, Aggregate level C). More studies are warranted to understand the relevance of the latter findings. It is worth noting that—in research with SARS-CoV-2—so far, little attention has been paid to the interactions of salt (NaCl) with proteins, receptors and enzymes. Such interactions occur *via* ionic/electrostatic bonding: they affect monomer/dimer/multimer configurations, equilibria and receptor binding. Describing the effects of salt, and so saline, on primary immune responses in human is beyond the scope of our analysis; these may, however, be relevant because SARS-CoV-2, its spikes and E-protein have been shown to affect ion channel activity (ENaC), to impair sodium and/or chloride transport and to induce membrane-potential silencing: these processes can be reversed by saline (20). For instance, the effects observed on the interferon genes in the proof-of-concept study in the hamsters (28) may be relevant to Omicron infection, as the Omicron spike confers enhanced infectivity and interferon resistance to SARS-CoV-2 in human nasal tissue (3). Earlier observations with another virus had shown that adding saline (50 mM) *in vitro* promoted type I interferon signaling in macrophages, thereby strengthening immune sensing and increasing antiviral resistance (80). During our analysis, we noted several signals from *in vitro* and *in vivo* experimental data with Omicron, indicating that saline may help to enhance the virus-neutralizing potency (Supplementary Tables S2, S3). This was corroborated by the effects of saline observed on infectivity or immune response in the single-rinse studies of saliva and nasal secretions (38, 39) and on inflammatory markers in clinical studies with SNI (Section 3.2.1). The relevance of the mechanisms vs. their primary rinse effect deserves further study. Our results are also consistent with a recent study in healthy volunteers revealing enhanced immunoglobulin-response-related fractions after 15 days of isotonic SNI (81). The new findings further support that saline does not act like an antiseptic, but rather through physiological mechanisms.

Overall, the findings add to the rationale for SNI and saline gargling in COVID-19, similarly to hygiene steps taken in common colds or URTIs. Coincidentally, after formal analysis for this review, a large RCT in primary care in the United Kingdom was published, evaluating, among other treatments, saline for the treatment of URTI. Similarly, as in the study of de Gabory et al. (47), this RCT reported the efficacy of SNI during the COVID-19 pandemic in patients with URTIs, irrespective of COVID-19 status. In the latter study, all patients had at least one comorbidity or risk factor increasing their risk of adverse outcomes due to respiratory illness upon enrolment (~2,900 patients per treatment group); one-fifth per treatment group received a COVID-19 diagnosis (82). In that study, 0.9% NaCl was used, “two sprays per nostril at the first sign of an infection or after potential exposure to infection, up to 6 times per day.” This large study thus confirms prior findings and supports our recommendation that SNI should be used at the earliest onset of symptoms.

The clinical results also revealed that the DVS of Omicron RNA material outlasts the duration of the rapidly resolving symptoms. There was a high variation in DVS between the studies, consistent with a recent meta-analysis (median values of viral shedding ranging between 5.83 and 17 days) (83).

Added agents to SNI and gargling deserve further study (Aggregate grade of evidence C, Summary Box 3). The reduction in viral load by adding molnupiravir to SNI was modest but real (46) (Section 3.2.3). Such a combination may offer advantages to patients in whom an antiviral is indicated, especially given that rebound after antiviral treatment is common (84). Potential benefits include fast and safe implementation of SNI and saline gargle following qPCR diagnosis, improved mucociliary clearance and local formation of antiviral hypochlorous acid (20). Saline gargling may also help to mask the bad taste caused by Paxlovid (85, 86). As antivirals are also expensive and not always reimbursed, further studies are warranted: faster negative conversion may help reduce hospitalization and emergency access, increase outpatient management, and reduce human and material healthcare resources, as recently suggested by a study, initiating SNI and gargling in patients presenting at an emergency clinic after positive qPCR-testing (60).

Early initiation of SNI and gargling is important; it was shown to affect the evolution of the viral load and symptoms, such as fever and smell and taste disorder (Aggregate grade of evidence B, Summary Box 2): irrigation and gargling likely complement each other given their adjacent anatomical sites of action. While saline gargling alone was insufficient for effectiveness against COVID-19 for reducing viral load (87), this review found that sufficient SNI volumes, which typically also irrigate the proximal throat, or in combination with gargling, may lead to reduced viral load and transmission (47), protect against taste/smell disorders (44), relieve fever (49), and respiratory symptoms (Section 3.2.2), reduce post-nasal drip and soothe a sore throat (33, 47, 88), as well as faster resume the ability to perform daily activities (47), and reduce the need for hospitalization (47, 60, 61).

SNI and saline gargling in hospitalized patients. While the risk for hospitalization is low with Omicron (89), our data from hospitalized patients with high Omicron viral loads (41, 42, 47, 49) or pneumonia (43) show that patients may benefit if (isotonic) SNI and saline gargling are started early; this is associated with increases in lymphocytes and a reduction in CRP (41, 46, 49, 68). The reduced risk for hospitalization or treatment escalation is supported by our earlier pre-Omicron assessment (20), and by a recent study of mixed variants comparing the effects of iso- and mild hypertonic SNI with gargling 4×/day: a lower hospitalization rate was reported with both saline strengths, while no deaths were observed with isotonic SNI plus gargling (60). Decreased hospitalizations and no deaths were also observed in a cohort of older adults with high BMI, mainly infected with the alpha-variant,who initiated isotonic SNI two times daily within 24 h of positive testing (61). Overall, isotonic saline seemed to be best tolerated, in particular in children, while corticosteroids, polyvidone iodine, adding molnupiravir, or herbal ingredients were associated with more AEs (Section 3.3.3).

The results on symptom relief are also in line with observations of benefits of SNI with gargling in common cold, flu and URTI (22, 82, 90–94), making this non-pharmacological hygiene intervention suitable for self-care. From a pathophysiological perspective, treatment with repetitive and large volumes of isotonic saline may be preferred: COVID-19 is characterized by exacerbated, aberrant mucus production to be cleared away (73, 95, 96). The pharmacodynamic basis for choosing an optimal strength and volume of saline for use in COVID-19 may thus differ from the expected actions of hypertonic saline (often favored) in more chronic sinonasal or chronic respiratory conditions, such as cystic fibrosis (97). Various techniques have been used (Table 2) (40, 41, 43, 98) and, albeit simple, are at best explained to, and ideally trained with, the patient either with a short film or by demonstration. In India, the technique of SNI, using a Neti pot or a saline infusion bottle, called Jala Neti, is an integral part of Yoga and Ayurveda interventions, established in various nasal disorders and has been used in COVID-19 (98, 100, 117).

**Table 2:** Techniques of SNI spray are instructed/trained to patients with COVID-19.

Device	Instructions
Nasal spray pump, releasing puffs (~0.2 mL /nostril) Cegolon 2022 (40)	Spray cylinder with nozzle: hypertonic solution based upon composition with 10% seawater, also enriched with xylitol and panthenol. Spray inside the nasal cavity. The patient’s head must be kept in upright position. The intranasal nozzle device is first inserted vertically inside the nostril to penetrate 5 mm into the airspace and subsequently inclined upon the horizontal plane for spraying. (No instructions on blowing the nose or what to do with drip into the mouth) Three times/day
Pressurizing nasal spray bottle, releasing volume during 10 s Liu 2023a (41) Liu 2023 (49)	Electric Children’s Seawater Nasal Cavity Sprayer (Aide Medical Co., Ltd., Guizhou, China): irrigation bottle to be filled with 10 mL of hypertonic (seawater) saline. The irrigation solution is applied to the patient in a seated position. The nozzle is directed into one nasal cavity, machine turned on with irrigation delivered for about 10 s. The patient is then told to blow out the rinse solution. The process is repeated for the other nasal cavity. Twice per day: once every morning and before bed
Spray bottle with nozzle Pantazopoulos 2023 (43) (hospitalized adult patients with severe pneumonia)	100 mL spray bottle with nozzle Blow nose before irrigation; gently tilt neck forward; slight tilt head to one side; insert the nozzle in the nostril parallel to the nasal septum; press firmly to squirt the solution; return to the upright position to allow the solution to work for some time; repeat in the other nostril. In case any solution ended up in their mouth, patients were advised to spit it out. The nozzle is washed with warm water and wiped dry after each use. Every 4 h for 16 h per day
Irrigation volumes in Neti pot or with saline infusion bottle Tyagi 2020 (103)	Jala Neti, SNI, nasal lavage, nasal douche aims at flushing out mucus, and debris and to clear the nose as to enhance nasal breathing. To perform over a recipient (sink, a bowl on a table or, in the shower or outside). Fill Neti Pot with warm water, add salt, taste and check suitable temperature for pouring into the nose. Place nose cone into the right nostril, sealing it inside after a few twists with slight pressure; avoid sniffing, swallowing, laughing or talking. Bend forward sidewise, so the left nostril becomes the lowest point of the nose. Keep on breathing from mouth, while the water comes through, coming out the left nostril after a few minutes. At this moment, breathing must be kept slowly and gently through the mouth. Blow out gently from both nostrils in order to clear water and mucus from the nose. Above-mentioned steps are to be repeated in the other nostril, if mucus blockage is still there, and over again until it clears entirely. If the blockage is not over, person needs to consult an ear, nose surgeon as to identify whether there is any blockage in the nose. Rastogi et al. (117) have proposed improvement in the Jala Neti procedure by adopting a Normal Saline infusion bottle of 100 mL for the purpose. This eliminates problems associated with the tonicity of the salt solution and its practical application while the person is on the move.
Chatterjee 2020 (68), online video	Chatterjee 2020 (68) Simple, typical plant pump sprayer, allowing to manually pump and to form mist by the nozzle, so to administer larger volume under mild pressure—easy to control till the nose is blown-out—see video links https://www.youtube.com/shorts/DuXREn8dML8 https://www.youtube.com/shorts/yG1ogCe6PX8
Parviz et al. 2020 (118), online video	Parviz et al. 2020 SWHF-ERNIG protocol, using self-prepared saline and a small sprayer bottle to generate volume to clear the nose https://www.youtube.com/watch?v=1yDgJ80hCoU

Benefits may also relate to reduced need for medicines (35, 54). SNI facilitates the reduced use of antibiotics, nasal decongestants, OTC antihistamines, antiseptics, mouth rinses, antitussives, and other nasal OTC remedies or sprays (23, 60, 99–102), which is relevant in view of their abuse upon self-medication—a problem which was already noted during the COVID-19 pandemic (103, 104). Moreover, recent antibiotic surveillance reports (105, 106) reported significant rebound of antibiotic use after the COVID-19 outbreak (characterized by lower antibiotic use due to less circulation of respiratory viruses), while we noted the lowest antibiotic consumption (ECDC) among the EU countries with traditional saline use or first-line recommendation of SNI for common colds (the Netherlands and Sweden) (105). The reduction of antibiotic use with the use of SNI has been recently confirmed in a large open-label RCT in patients with URTI, one-fifth of them diagnosed with COVID-19 (82).

The outcomes of prophylaxis studies are less definitive (Aggregate grade of evidence: C; Summary Box 4) but deserve mention as signals for future research. Early data suggest that SNI helps to prevent transmission, but a combination with strict use of PPE is required. From a daily use perspective by healthcare workers, consistently wearing PPE, and using SNI two times daily and upon mask changes, may already help to load up masks with more salt from their exhaled condensate: salt on mask materials has been found to better filtrate and inactivate viral particles (107, 108). The findings on prophylaxis are consistent with the proof-of-concept study, where the transmission in the recipient hamsters was significantly reduced, but not fully suppressed (37). Moreover, considering (a) the dynamics of rinsing (removing virus and infectious mucus) (20, 73), (b) the outcomes of single-rinse studies (no immediate neutralizing effect unless 0.5–6 h after SNI) (38, 39), and (c) the outcomes regarding transmission apparently also being affected by the strain (28, 47), repetitive rinsing of mouth & nose combined is likely to perform better to remove virus and mucins efficiently, particularly in the home setting where PPE is often less stringently applied.

From a pandemic preparedness perspective (the response to prepare upon the appearance of a new virulent respiratory infectious agent), saline solution should not be tested as an antiseptic (which led to its ban as a myth during the past pandemic), but should rather be tested for (1) its rinse potential: its capacity to remove infectious virus, vesicles, and mucins from nasal epithelium (74) (2), its potential to enhance the virus neutralizing action in saliva and nasal secretions (38, 39), and (3)—taking the concept physicochemical further—to aggregate virus at its iso-electric point, toward formation of larger particles that are likely better filtered out by masks. Addition of (concomitant) antimicrobial agents to SNI(G)—be it antiseptics, essential oils or other—deserves careful study, on the one hand, of the PCR test (potentially inhibited, so inducing false negatives), on the other hand for the tolerability; potential detrimental shifts in oronasal microbiota should also be considered (28). As summarized in Table 3, several questions remain to be addressed, yet these do not prevent its safe application for self-care of COVID-19, overlapping with URTI, as evidenced from the French RCT, reaching comparable results when analyzing all patients with URTI, COVID-19 inclusive, vs. COVID-19 and diagnosed URTI alone (47). The recently published large RCT assessing SNI in the United Kingdom adds evidence for such an approach (82). Moreover, some have proposed that SNI (Jala Neti) may have a certain role to play in COVID-19-associated complications, and so, is at best done at home until definitive care is available: this was for instance proposed for COVID-19 associated mucormycosis (114, 115), yet still needs further validation for its utility in this challenging, complex condition (116).

**Table 3:** Questions relevant to saline, COVID-19, and URTI to be addressed in the future.

***Can fast initiation of saline SNI / gargling be of benefit for patients presenting at the emergency department, and so reduce the hospital burden of the disease?*** This question has been addressed with promising outcomes, but unfortunately, with small patient numbers, and/or lacking adequate controls. The absence of mortality in 2 studies with physiologic (0.9%) saline needs confirmation in larger surveys.
***Can saline reduce the need of expensive antivirals and reduce rebound?*** To note, many may not have access to these expensive antivirals, that often are not reimbursed, or even not available.
***Can saline reduce transmission reliably if combined with mask wear?*** Develop a survey protocol and repeat the results reported by Chinese during Omicron infection in a controlled fashion. Consider PPE (mask use, hand hygiene), SNI and gargling, volume and frequency of use, and ideally at a new outbreak, also the saline formulation vs. characteristics of the virus strain and its inactivation in nasal fluid or saliva.
***Can saline reduce or prevent Long-COVID-19 or other associated complications?*** This question can only be answered by long-term follow-up of large studies.
***Can saline contribute to respiratory pathogen pandemic preparedness (the latter action proposed by WHO)?*** SNI was discouraged at the start of the pandemic; research findings since then show that SNI and gargling are associated with benefits. Development of study protocols are warranted, to be ready at start of a pandemic.

### 4.1 Limitations

Limitations of the current study are those consistent with the PRISMA-based systematic review of literature. Limitations of the reviewed studies include small sample size and limited participant blinding capacity (21). The studies had heterogeneous trial designs and outcome variables, which is not surprising given that they were not typically part of large initiatives but rather performed by investigators in local clinics or practices. These studies were generally insufficiently powered to detect differences in outcomes, such as hospitalization. Smaller studies carry some risk of bias because of the impact of co-morbidities on COVID-19 outcomes, while symptoms can be highly variable (109). The study design by Jing et al. overcomes part of the blinding issue by adding a double-blind-double-dummy comparator group and engaging pharmacists for randomization and nurses for training on SNI (44). Given that Omicron infection is an important public health condition, but symptoms are mild and can also resolve quickly in some patients (40, 41, 49, 50, 94, 110, 111), large RCTs may be needed to detect significant changes with SNI and gargle. Moreover, while ideally health authorities should make funds available to study this hygiene measure, such large studies may face difficulties for finding significant results, due to the less stringent application of hygiene interventions in larger studies in the community, as has been the case with studies of other hygiene measures, such as mask wear (112). However, similar to other sinonasal conditions (113), our review allows one to consider what can realistically be achieved clinically in discussion with patients while providing routine care, as well as what can be achieved by using SNI for self-care of COVID-19.

## 5 Conclusion

Consistent with our original report (20), the benefits identified in Omicron studies and pre-Omicron studies support the finding that combined SNI with saline gargling, using solutions prepared with clean water, and using a clean nasal cup, spray, or squeeze bottle, can be early and safely recommended in mild-to-moderate COVID-19 to reduce viral loads and to help relieve throat pain, cough, nasal and/or respiratory symptoms—analogous to the pragmatic approach taken with SNI in common cold, URTI or other respiratory conditions. Combined antiviral/SNI treatment in patients at risk for progression to severe disease requires further study. From a pandemic preparedness perspective, SNI with saline gargle deserves consideration as routine hygiene in COVID-19, and as reasonable recommendations for prevention and treatment of common cold or URTI. Further study in adequately funded large trials is needed to optimize protocols and better understand outcomes.

## Data Availability

The original contributions presented in the study are included in the article/Supplementary material; further inquiries can be directed to the corresponding author.
