# Supplementary material for: Saline nasal irrigation and gargling in COVID-19: Part II. Outcomes in Omicron and risk–benefit for self-care
Source: Front Public Health. 2025 Aug 20;13:1462286. doi: 10.3389/fpubh.2025.1462286 (PMC12406277; doi:10.3389/fpubh.2025.1462286)
Supplement: Supplementary file 1 [file Supplementary_file_1.pdf]

Supplementary material : *Saline nasal irrigation and gargling in SARS-CoV-2 Omicron infection*

Methods 1
2.1. Systematic Searches 1
2.2 Analysis of symptom outcomes of RCTs pre-Omicron 2
2.3. Data processing 2

Results
Figure S1. Flow diagram of search results & tabulation 4
Table S1. Reasons for exclusion of studies of SNI 5
Table S2. In vitro effects of 0.9% saline (dilution) relevant to Omicron 9
Table S3. Effect of Nasal Saline Irrigation (SNI) or gargling in Omicron infection: proof-of-concept study in the Golden Syrian hamster 10
Table S4. Effect of Nasal Saline Irrigation (SNI) or gargling in Omicron infection: single-rinse randomised clinical trials 11
Table S5. Effect of Nasal Saline Irrigation (SNI) on viral shedding and symptoms: Clinical treatment studies: 12
Table S6. Effect of Nasal Saline Irrigation (SNI) and/or gargling on COVID-19 symptoms and tolerability: Pre-Omicron RCTs: 18
Table S7. Bias assessment : studies relevant to mechanism of action (studies Table 1) 22
Table S8. Bias assessment: studies on viral shedding and symptoms (studies in Table 2) 23
Table S9. Bias assessment: RCTs on COVID-19 symptoms and tolerability, pre-Omicron 25
Table S10. Assessment of aggregate level of evidence 26
Table S11. PRISMA checklist [no meta-analysis] 29

Supplementary information to:

Methods:

This review with systematic search strategies is the extension of a pro bono initiative by a multidisciplinary network ; this review study has not been registered in a trial data base. The review protocol followed the method used for studies on oronasal saline, earlier published (Huijghebaert S, Frontiers 2023), yet adapted to Omicron.

S2.1. Systematic searches:

Primary systematic searches were performed combining 'saline' or 'seawater' or 'nasal irrigation' or 'gargling' and 'Omicron' as MeSH search terms (=primary searches) on PubMed. We also performed secondary searches combining the words 'saline' with 'COVID-19' and dates '2022' or '2023', and studies relevant to SNI were retained for reviewing, which was also regularly repeated broader on internet (Figure 1S) and summarised in Figure 1. Searches and update were closed 22/12/2023.

Exclusion and inclusion criteria were the same as in the former review (Huijghebaert et all. Frontiers 2023) with the following alterations or additional criteria:

- As no separate search was performed for mechanisms of action, relevant in vitro and in vivo (proof-of-concept) studies were retained. The in vitro studies were tabulated in Table 2S, if relevant, but these are not the focus of this review.
- Only experimental or clinical studies referring to Omicron, of being performed in the local time frame starting at surge of Omicron (usually started December 2021) were retained.
- If any doubts, or studies were subsequently excluded for other reasons, these were listed in Table 2S with their reasons for exclusion.

- As focusing on self-care in mild-to-moderate COVID-19, presenting at home, studies using inhalation (nebulized saline) in severe COVID-19 (ventilation) were not retained. Despite the allegation that saline nebulization is an aerosol generating procedure increasing the risk of transmission, has been refuted (Huijghebaert 2023), inhalation is still considered in many countries a procedure at risk of enhancing transmission. Yet, as dynamics may differ in such patients compared to uncomplicated mild-to-moderate COVID-19, such studies were not retained.

As upon finalization of the manuscript 2 more relevant studies were published, an additional check was done on PUBMed, identifying in total 3 more studies, 2 of which were retained (added to the schema) , one rejected and added to table 2S) .

S2.2. **Analysis of symptom outcomes of RCTs pre-Omicron**

For all Omicron studies, the effect on symptoms was listed in the Tables, including:

- time to symptom resolution or symptom relief (TTSR)
- any other information on symptoms outcomes, if available.

We further evaluated the outcome on symptom in Omicron infection, in comparison with the results from randomised controlled studies (RCTs) prior to the surge of Omicron (further referred to as pre-Omicron studies). These articles provided sufficient data for such evaluation, by including also studies using saline intervention as placebo versus a comparator group. The list was updated with new articles identified pre-Omicron, using the described search strategy (Huijghebaert 2023) [One more new pre-omicron RCT identified]. The pre-omicron RCTs are listed in Table 8S. Case-control studies were not listed. Also RCT only assessing smell and taste dysfunction were not retained for this tabulation. In total 8 studies were retained, in which SNI was compared to controls (N=5) or served as placebo (N=3). All but one additional RCT (Siregar 2022) originated from the former evaluation (Huijghebaert 2023).

As studies were heterogenous in symptomatic assessments, both with regard to parameters and methods used, following outcomes were listed, apart from TTSR:

- % patients with symptom resolution (PSR)
- symptom severity (SS) change
- improvement of symptoms (ImS), and
- any other information on symptoms, if available.

The assessment method was also tabulated, e.g. visual analogues scale (VAS), or adapted Wisconsin Upper Respiratory Symptom Survey (WURSS). To assess tolerability, the most frequent adverse events or adverse effects (AEs) were tabulated.

S2.3. Data processing

Data were collected by the coordinator, and at least 2 reviewers screened each record revising the retrieved data from the articles. Data were analysed/reported per type of patients or treatment, and study design, as to reveal the heterogeneity among study results, as well as whether saline served as placebo/control or was the active intervention studied. Missing results/treatment groups (so possible reporting bias) were mentioned if applicable. Assessments of certainty in the body of evidence was evaluated for each outcome by also taking the pre-Omicron data into account, and by in vitro results that were identified on Omicron through our systematic searches (listed in Table 2S)

As RCTs used different trial designs and were often limited in patient number, while bias assessment is difficult in case of SNI, the bias assessment was performed thereby also considering the rationale for choices for each of the studies (**Tables 4S-6S)**. To note: saline was sometimes used as a placebo. In addition, SNI cannot be blinded due to its salty taste and irrigation volume/technique, and SNI requires training at the start of a study. Data were collected and processed narratively, as the different study

designs made collation of the data difficult, so no pooled or meta-analysis was performed. Pre-omicron studies evaluating SNI only for COVID-19 smell and taste disturbances were not analysed and will be part of a separate evaluation, in light of the new findings for Omicron infection by Jing et al. 2023.

3. Results

3.1. Flow diagram of search results & Tabulation

For search terms and search strategy and flow diagram, see Figure 1S: as extensive parallel searches were performed difficult to present in one Figure, the main findings are summarised in Figure 1 of the main article.

Reasons for exclusion of studies of SNI (N=28) are tabulated Table in 1S.

In vitro outcomes were tabulated in Table 2S. These included in vitro effects of 0.9% saline on or *vs* saliva relevant to Omicron ,relating to antibody-antigen reaction, rinse effect and infectivity (N=6)

2.2. Consolidated overview of Omicron study material

In total, 14 relevant studies were retrieved, 12 up to 2023, 2 more in 2024. One experimental proof-of-concept study with isotonic SNI (0.9% NaCl) (Yuan 2022) and two single-dose RCTs with 0.9% as placebo, nasal drops (Imsuwansri 2023) or gargle (Bonn 2023) revealed insights on mechanism of action (Table 1). Relevant in vitro studies (n=7) identified during the systematic screening are reported in Table 2S.

Ten clinical studies assessed repeated SNI in patients with Omicron (Cao 2022, Cegolon 2022, de Gabory 2024, Liu 2023a, Liu 2023b, Lin 2023, Jing 2023, Pantazopoulos 2023, Yan 2024, Zou 2022), one SNI plus gargling (Jing 2023), and SNI plus PVI after hospital discharge (Liao 2023): see Table 2. Two studies were already covered in the first review (Zou 2022, Cao 2022). Five studies were non-blinded RCTs: four in adults (Zou 2022, Cegolon 2022, Pantazopoulos 2023, de Gabory 2024) and one paediatric study (Lin 2023). One RCT was a blinded RCT on the prevention of smell and taste dysfunction, comparing SNI plus saline nasal spray and saline mouth rinse, with SNI plus budesonide nasal spray plus chlorhexidine mouth rinse, while also comparing in randomised manner with controls (Jing 2023). Six studies assessed the effect on viral load versus controls (Cao 2022, Cegolon 2022, de Gabory 2024, Liu 2023a, Liu 2023b, Lin 2023), while one RCT compared molnupiravir with SNI versus SNI alone (Zou 2022). Patients received standard of care [generally anti-flu Chinese granules in 4 studies (Liu 2023a, Liu 2023b, Lin 2023, Zou 2022)] while one study assessed OTC medication consumption to control symptoms (Cegolon 2022). There were two case-control studies, one prospective study reporting on development and duration of fever comparing to 3 control groups who were not rinsing the nose (Yan 2024), and one prospective study assessing rebound following application of NSI + polyvidone iodine (PVI) after hospital discharge. Two studies also reported on prophylaxis (Cao 2022, Yan 2024) and one on household transmission (de Gabory 2024). Three more studies (not listed in the tables), are reported for inflammatory parameters and hospitalization risks (Beigmohammadi 2023, Chatterjee 2023, Espinoza 2023): for the reasons of not tabulating, see Table 2S.

Studies varied in baseline parameters at recruitment, disease severity, design and saline strengths, compositions, dosing frequency or volume, rendering the pooling of data inappropriate. Therefor study results were reported narratively, and conclusions on outcome for use in clinical practice drawn.

Figure S1: Results from searches up to 22.12.2023 | **After removing duplicates: N=12**

Primary searches

Search "Omicron AND Saline" (up to 22.12.2023): n= 22
/Excluded: n=13 (6 vaccines, 2 sampling/diagnosis, 4 other not relevant to SNI; 1 general discussion)
\Included: n=3 in vitro studies relating to/relevant to omicron (Table 2S)
\Included: n=6 clinical studies

Search "Omicron AND Seawater" (up to 22.12.2023): n= 5
/Excluded: n=3 (other scope, not relevant to SNI)
\Included: n=2 clinical studies

Search "Omicron & nasal irrigation" (up to 22.12.2023): n=15
/ Excluded n=10 (3 vaccine, 4 sampling/diagnosis, 3 various other)
\ Included : n=5 clinical studies, all covered by other primary searches

Search "Omicron & gargling" (up to 22.12.2023): n=9
/Excluded n=6 (4 sampling/diagnosis, 2 various other)
\Included: n=3 in vitro studies relating to/relevant to omicron (Table 2S)

Secondary searches

Search "COVID-19 AND saline AND 2022" (up to 22.12.2023): n=199
20 out of 199 eligible for SNI or gargling/saline inhalation/mechanisms with specific reference to omicron
/ 12 Clinical studies not eligible as performed prior to omicron surge, or studying other aspects (Table 2S)
\Included: 3 in vitro studies relating to/relevant to omicron (Table 2S)
\Included: 4 Clinical studies overlapping with other searches
\Included: 1 Clinical study (Cegolon 2022)

Search "COVID-19 AND saline AND 2023" (up to 22.12.2023): n=138
22 out of 138 eligible for SNI or gargling/saline inhalation/mechanisms with specific reference to omicron:
/12 Clinical studies not eligible as performed prior to omicron surge, or studying other aspects (Table 2S)
\Included : 3 in vitro studies relating to/relevant to omicron (Table 2S)
\Included : 5 Clinical studies overlapping with other searches
\Included: 2 RCT (smell-taste disorders Jing 2023 + rebound Liao 2023)

Internet searches & Personal communications: n=7
/Excluded (Table 2S): n=4
\Included: n=3 (Yuan 2022, Bonn 2023, Zou 2022) [all PubMed listed,not identified by above searches]

N=9

7 studies in adults

- **1 mechanism-of-action RCT**
- **3 RCTs**
- **1 quasi-experimental study**
- **1 hospital + prophylactic study**
- **1 matched control (assessing rebound after 1-week saline following hospital discharge)**

2 studies in children

- **1 RCT**
- **1 quasi-experimental study**

[7 in vitro studies relevant to mechanism of action]

+

N=3 (Adults)
1 experimental study
1 single-gargle RCT
1 RCT combined with antiviral

Table S1. Reasons for exclusion of studies of SNI (n=28)	
First author	**Reason for exclusio**n
Alsaleh 2024	Major reason: RCT <10/group: n= 5 (PVI) – 6 (NSI) – 8 (control) subjects per group.<br>Other reasons:<br>• Mouth rinse (20mL) was 10 sec only, too short for saline to be relevant (2x/day)<br>• Survival analysis favours PVI, yet: (1) no information on duration before enrolment (≤72 h of developing symptoms): essential info for such a small study: (2) only 4 PVI plotted (cross-mark in survival graph: dropout?), the 6 NSI participants plotted do not match realistic patient numbers in the 2 last steps<br>• Accuracy of Ct values is questionable; mean Ct on day4 worsened from 23 to a 15 (± 11.7) with NSI despite 2 participants already PCR(-) on Day 4 implicating that all 6 others would have had CTs between 10-12 , while differences between Ct values did not reach significance across the 3 groups (*p*-values 1 - 0.07 - 0.83, resp.); outcomes are moreover discordant with the SNOT score evolution – P = 0.08 (best response with saline); WURS score = presented as resulting in a difference with PVI vs saline, yet was not significantly different (p = 0.75).<br>• No care was taken to separate sampling moment from use/administration (PV inhibiting qPCR test)
Aref 2022	Ivermectin versus saline nasal spray for post-COVID-19 anosmia: RCT, however only small puff volume used not representative of SNI (twice a day).
Batioglu-Karaaltin 2023	RCT performed September and October 2021 (n=30/group). Controls, versus isotonic SNI, versus isotonic SNI + polyvidone iodine (PVI)1%, versus hypertonic SNI +PVI 1%. Conclusion: significant effects for PVI 1% added to isotonic or hypertonic SNI (but not isotonic SNI) on viral load compared to controls. Yet, many potential biases:<br>• Volumes administered by the nozzles and frequency not disclosed ('continuous nasal spray with sun-proof white tubes'). Jump from isotonic to hypertonic SNI saline in combinations with PVI. Favipavir was given to all subjects presenting at emergency department, albeit subjects were mostly asymptomatic at baseline (median scores: 0 for all symptoms);<br>• No information on co-morbidities or further symptom evolution.<br>• Major bias = baseline viral loads being at least 10x lower in isotonic saline group (so reducing the magnitude of "change in viral load" in disfavour of saline) , while the highest loads to start in the 2 PVI groups (allowing the highest magnitude in reduction); other major problem: the loads in text do not match those in the Fig.
Baxter 2022	RCT performed September 24 and December 21, 2020
Beigmohammadi 2023	ICU study (December 2021 to February 2022, unclear if Omicron, while not relevant to mild-to-moderate disease and selfcare):<br>RCT in ICU admitted severely ill, refractory patients with severe pneumonia treated during masking for oxygen support were administered inhalation of hypertonic saline (HS) (5%, 10 mL nebulized 4 times daily) or distilled water (10 mL 5% NaCl, every 6 hours for 5 days). Results:<br>• No significant differences between the study groups in terms of intubation rate, length of hospital stays, or length of stay in the ICU.<br>• TNF-α, IL-6, Na, ESR levels, leukocyte count, and PO2 significantly improved with HS.<br>• Serum TNF-α and IL-6 increased in controls vs decreased with HS (*P* < 0.0001, *P* = 0.003, resp.). CRP levels slightly increased with HS while remained unchanged in the water group (*P > 0.05*).<br>• Mortality: numerically higher with HS (11/30; 9/30 with water). Baseline data reveal more than twice diabetes mellitus co-morbid cases in the HS group (11/30), not corrected for, versus controls (5/30).<br>Overall, 5-day HS inhalation in refractory patients did not ameliorate CRP-values in comparison with sterile water, despite many other parameters improving significantly with HS inhalation. As twice as many patients in the HS group had co-morbid diabetes mellitus, this may have affected mortality in disfavour of saline.
Chalageri 2022	RCT performed September 2020 to February 2021.

Chatterjee 2023, personal communication (co-author)	Open-label RCT in ICU patients with Omicron infection and severe pneumonia, in need of oxygen support upon hospital admittance (performed 2022-2023): SNI with isotonic saline was performed at least 4 times daily versus controls (20 mL every 3 hours).<br>Preliminary findings:<br>• Daily isotonic SNI in addition to SOC was found to stabilize or decrease CRP and increase lymphocyte counts.<br>• Neutrophil /lymphocyte ratio decreased in the half of the patients, no longer in need of respiratory support escalation.<br>(Results by personal communication by Chatterjee 2023).
Chuayruksa 2023	Retrospective analysis case control study: Saline irrigation protective in HCW, either tested by qPCR or antigen-antibody tests (or both):<br>• qPCR-test: 6.1% qPCR-positive (14/230 cases) using saline versus 69.5% (324/466 cases) not using SNI (P < 0.003 for Odds ratio)<br>• Antigen test: 24.0% positive (6/25) versus 42.1% not using SNI (284/671 cases; P < 0.001 for Odds ratio)<br>To note: saline use may thus be a risk for false positive with (some?) antigen tests: this is nor surprising if these contain less selective cross-reactive antibodies, in view of the efficacy of saline identified by review in promoting antigen-antibody binding.<br>– Yet pre-omicron: 696 samples were retrieved from databases of 43 files reports in August 2021.
Colado Simão 2023	CT performed November 1, 2020, to February 1, 2021.
Delić 2022	RCT, performed October 2020 and June 2021.
Espinoza 2023	Mixed study design: RCT for low- (N=27) vs high saline (N=28) in warm water, SNI plus gargling four times a day for 14 days; compared with a group of matched controls (matched reference population, yet not fully matched for race), admitted to emergency, and treated in period between 2020 and 2022 (so, bias possible by difference in distribution of variants).<br>• Hospitalization following 0.9% saline: 18.5%; with high (=2.3%) saline: 6%; reference population: 58.8% ($P < 0.001$).<br>• Pneumonia following 0.9% saline: 14.8%; with high (=2.3%) saline: 17.9%; reference population: 28.2% ($P = 0.25$).<br>• Antiviral or monoclonal antibody treatment (cumulative) following 0.9% saline: 11.1%; with 2.3% saline: 7%; reference population: 23.7% (P = 0.58-<0.01).<br>• ICU following 0.9% saline: 2%; with high (=2.3%) saline: 1%; reference population: 3.53% ($P = 0.99$).<br>• Mechanical ventilation: following 0.9% saline: 0%; with high (=2.3%) saline: 3.6%; reference population: 2.06% ($P = 0.99$).<br>• Death: following 0.9% saline: 0%; with high (=2.3%) saline: 7.1%; reference population: 5.51% ($P = 0.99$).
Esther 2022	RCT. First publication date on Research square; May 14, 2021: prior to Omicron surge.
Fleming 2023	Report of the results (number of treatments, orders, and full-time employees) associated with administering nebulized 3% hypertonic saline plus N-acetyl cysteine (HS/NAC) from a policy "de-implementing" HS/NAC nebulizer treatment in Wisconsin hospitals, to reduce the burden for the staff. HS/NAC was considered a low-value care, as being a practice lacking evidence-based efficacy while burdening health care workers. Effects of "de-implementing" the nebulizer treatment on patient outcomes were not assessed.
Gangadi 2022	Open-label survey June 2021 to March 2022.
George 2022	RCT period May–June 2021 when the delta variant was predominant
Gupta 2023	RCT March 15 and August 31, 2021, on role of SNI plus theophylline for treatment of COVID-19–related olfactory dysfunction.
Hautefort 2023	Period/variant not mentioned.<br>Hyposmia: SNI versus SNI + budesonide: no efficacy of adding budesonide.
Johnson 2023	Testing of lung hyperreactivity.
Karpishchenko 2023	Study reporting the experience with olfactory training with a set of essential oils. Aromatherapy was preceded by SNI with isotonic saline solution.

Mohamad 2022	RCT of various treatments and SNI (=controls) on smell dysfunction, performed January 1, 2021 to February 28, 2021. Yet, the so-called 'normal' saline appears not be only 0.2% NaCl.
Natto 2022	RCT mouth rinses vs saline control, performed June to July 2021.
Pantazopoulos 2022	Study performed June 1st to August 31st, 2021 (prior to Omicron).
Sevinç Gül 2022	Manuscript received on April 15, 2022 (period unclear).
Soler 2022	RCT, xylitol vs saline nasal spray: no period mentioned but 2020-dated protocol number, while the publication was accepted July 2022.
Tanni 2023	No SNI, but dry nasal spray of 2.0 mg NaCl powder, particles sizes between 1–10 µm: cough frequency after 10 days BREATHOX® use was reduced when compared with SOC (*P < 0.034*).
Tragoonrungsea 2023	Randomised controlled study, July 2021 to December 2021.
Yildiz 2022	Epub July 10, 2021, which is prior to the Omicron surge date.
Zarabanda 2022	Publication on October 25, 2021, which is prior to Omicron surge date.
Zhang 2023	Assesses cough prevention upon extubation (rather than assessment of the infection).

References Table S1:

Alsaleh, S., Alhussien, A., Alyamani, A. et al. Efficacy of povidone-iodine nasal rinse and mouth wash in COVID-19 management: a prospective, randomized pilot clinical trial (povidone-iodine in COVID-19 management). BMC Infect Dis 24, 271 (2024). https://doi.org/10.1186/s12879-024-09137-y

Aref ZF, Bazeed SEES, Hassan MH, Hassan AS, Ghweil AA, Sayed MAA, Rashad A, Mansour H, Abdelmaksoud AA. Possible Role of Ivermectin Mucoadhesive Nanosuspension Nasal Spray in Recovery of Post-COVID-19 Anosmia. Infect Drug Resist. 2022 Sep 19;15:5483-5494. doi: 10.2147/IDR.S381715.

Batioglu-Karaaltin A, Yigit O, Cakan D, Akgul O, Yigit E, Yilmaz YZ, Cakir KB, Ciftci G, Boyoğlu NS, Cagliyan A, Can E, Dikme O, Hacioglu Y, Balkan II, Enver O, Ozdogan HA. Effect of the povidone iodine, hypertonic alkaline solution and saline nasal lavage on nasopharyngeal viral load in COVID-19. Clin Otolaryngol. 2023 Jul;48(4):623-629. doi: 10.1111/coa.14056.

Baxter AL, Schwartz KR, Johnson RW, Kuchinski AM, Swartout KM, Srinivasa Rao ASR, Gibson RW, Cherian E, Giller T, Boomer H, Lyon M, Schwartz R. Rapid initiation of nasal saline irrigation to reduce severity in high-risk COVID+ outpatients. Ear Nose Throat J. 2022 Aug 25:1455613221123737. doi: 10.1177/01455613221123737.

Beigmohammadi MT, Amoozadeh L, Naghibi N, Eslami B, Fattah Ghazi S, Javaherian M, Khajeh-Azad MA, Tabatabaei B, Abdollahi A, Nazar E. Effects of nebulized hypertonic saline on inflammatory mediators in patients with severe COVID-19 pneumonia: A double-blinded randomized controlled trial. Sci Prog. 2023 Oct

Chalageri VH, Bhushan S, Saraswathi S, Ranganath TS, Rani VD, Majgi SM, Vijay K, Hema MS, Sanadi SL, Nasreen PM, Shoyaib KM, Partheeban I, Vanitha B, Souza ND, Vaddatti JS. Impact of Steam Inhalation, Saline Gargling, and Povidone-Iodine Gargling on Clinical Outcome of COVID-19 Patients in Bengaluru, Karnataka: A Randomized Control Trial. Indian J Community Med. 2022 Apr-Jun;47(2):207-212. doi: 10.4103/ijcm.ijcm_804_21.

[Personal commmunication] Chatterjee 2023, Omicron study. personal communication.

[Internet retrieved] Chuayruksa. N, Phakdeekul W, Kedthongma W. Oral rinse, nasal irrigation, and risk factor of COVID-19 screening. Journal of International Dental and Medical Research 2023;16 (3) :1227 – 1231. https://www.jidmr.com/journal/wp-content/uploads/2023/09/46-D23_2820_Wuttiphong_Phakdeekul_Indonesia.pdf

Colado Simão AN, Perugini Stadtlober N, Stinghen Garcia Lonni AA, Venâncio LM, Lerner Trigo G, de Souza Cassela PLC, Mastellini Sanches Silva T, De Fátima Oliveira Hirth Ruiz M, Batisti Lozovoy MA, Tano ZN, da Fonseca Orcina B, Vieira Vilhena F, da Silva Santos PS. Effect of phthalocyanine oral and nasal antiseptic solutions on the infectivity of SARS-CoV-2 in patients with COVID-19: a randomized controlled trial. Ger Med Sci. 2023 Jun 23;21:Doc07. doi: 10.3205/000321

Delić N, Matetic A, Domjanović J, Kljaković-Gašpić T, Šarić L, Ilić D, Došenović S, Domazet J, Kovač R, Runjić F, Stipić SS, Duplančić B. Effects of Different Inhalation Therapy on Ventilator-Associated Pneumonia in Ventilated COVID-19 Patients: A Randomized Controlled Trial. Microorganisms. 2022 May 28;10(6):1118. doi: 10.3390/microorganisms10061118.

[Internet retrieved] Espinoza S, Trauffler L, Shamshirsaz A, Shamshirsaz A, Espinoza A, Espinoza J, O'Brien A. Double blind randmised controlled trial of saline solution gargling and nasal rinsing inSARS-COV-2 infection. Annals of Allergy, Asthma & Immunology 2023; 131 (Supplement 1): S82. https://doi.org/10.1016/j.anai.2023.08.245

Esther CR Jr, Kimura KS, Mikami Y, Edwards CE, Das SR, Freeman MH, Strickland BA, Brown HM, Wessinger BC, Gupta VC, Von Wahlde K, Sheng Q, Huang LC, Bacon DR, Kimple AJ, Ceppe AS, Kato T, Pickles RJ, Randell SH, Baric RS, Turner JH, Boucher RC. Pharmacokinetic-based failure of a detergent virucidal for severe acute respiratory syndrome-coronavirus-2 (SARS-CoV-2) nasal infections: A preclinical study and randomized controlled trial. Int Forum Allergy Rhinol. 2022 Sep;12(9):1137-1147. doi: 10.1002/alr.22975. Epub 2022 Jan 31.

Fleming K, George JL, Bazelak SJ, Roeske JA, Biggs AD, Landry CM, Lipchik RJ, Truwit JD. Optimizing Respiratory Therapy Resources by De-Implementing Low-Value Care. Respir Care. 2023 May;68(5):559-564. doi: 10.4187/respcare.10712

[Internet retrieved] Gangadi M, Georgiou S, Moschotzopoulou E, Antronikou T, Kainis E, Alevizopoulos K. Efficacy and safety of a hypertonic seawater nasal irrigation solution containing algal and herbal natural ingredients in patients with COVID-19. Eur Rev Med Pharmacol Sci. 2022 Dec;26(2 Suppl):112-123. doi: 10.26355/eurrev_202212_30495.

George CE, Scheuch G, Seifart U, Inbaraj LR, Chandrasingh S, Nair IK, Hickey AJ, Barer MR, Fletcher E, Field RD, Salzman J, Moelis N, Ausiello D, Edwards DA. COVID-19 symptoms are reduced by targeted hydration of the nose, larynx and trachea. Sci Rep. 2022 Mar 29;12(1):4599. doi: 10.1038/s41598-022-08609-y.

Gupta S, Lee JJ, Perrin A, Khan A, Smith HJ, Farrell N, Kallogjeri D, Piccirillo JF. Efficacy and Safety of Saline Nasal Irrigation Plus Theophylline for Treatment of COVID-19-Related Olfactory Dysfunction: The SCENT2 Phase 2 Randomized Clinical Trial. JAMA Otolaryngol Head Neck Surg. 2022 Sep 1;148(9):830-837. doi: 10.1001/jamaoto.2022.1573.

Hautefort C, Corré A, Poillon G, Jourdaine C, Housset J, Eliezer M, Verillaud B, Slama D, Ayache D, Herman P, Yavchitz A, Guillaume J, Hervé C, Bakkouri WE, Salmon D, Daval M. Local budesonide therapy in the management of persistent hyposmia in suspected non-severe COVID-19 patients: Results of a randomized controlled trial. Int J Infect Dis. 2023 Nov;136:70-76. doi: 10.1016/j.ijid.2023.08.022.

Johnson NM, Saunders MJ, Womack CJ, Kurti SP. The impact of COVID-19 on pulmonary function and airway reactivity after recovery in college-aged adults. Appl Physiol Nutr Metab. 2023 Jul 1;48(7):507-513. doi: 10.1139/apnm-2022-0410.

Karpishchenko SA, Lavrenova GV, Baranskaya SV, Zhamakochan KC. [Olfactory impairment in patients of the older age group with COVID-19 in the acute period and in the period of convalescence.]. Adv Gerontol. 2023;36(3):339-345. Russian.

Mohamad SA, Badawi AM, El-Sabaa RM, Ahmad HM, Mohamed AS. Study of Different Local Treatments of Post COVID-19 Smell Dysfunction. Iran J Otorhinolaryngol. 2022 Nov;34(125):281-288. doi: 10.22038/IJORL.2022.58339.3012.

Natto ZS, Bakhrebah MA, Afeef M, Al-Harbi S, Nassar MS, Alhetheel AF, Ashi H. The short-term effect of different chlorhexidine forms versus povidone iodine mouth rinse in minimizing the oral SARS-CoV-2 viral load: An open label randomized controlled clinical trial study. Medicine (Baltimore). 2022 Jul 29;101(30):e28925. doi: 10.1097/MD.0000000000028925.

Pantazopoulos I, Chalkias A, Mavrovounis G, Dimeas I, Sinis S, Miziou A, Rouka E, Poulas K, Gourgoulianis K. Nasopharyngeal Wash with Normal Saline Decreases SARS-CoV-2 Viral Load: A Randomized Pilot Controlled Trial. Can Respir J. 2022 Sep 27;2022:8794127. doi: 10.1155/2022/8794127.

Sevinç Gül SN, Dilsiz A, Sağlık İ, Aydın NN. Effect of oral antiseptics on the viral load of SARS-CoV-2: A randomized controlled trial. Dent Med Probl. 2022 Jul-Sep;59(3):357-363. doi: 10.17219/dmp/150831.

Soler E, de Mendoza A, Cuello VI, Silva-Vetri MG, Núñez ZH, Ortega RG, Rizvi SA, Sanchez-Gonzalez M, Ferrer G. Intranasal Xylitol for the Treatment of COVID-19 in the Outpatient Setting: A Pilot Study. Cureus. 2022 Jul 23;14(7):e27182. doi: 10.7759/cureus.27182.

Tanni S, Wehrmeister F, Prudente R, Damatto F, Breda Neto C, Oliveira L, Pagan L, Gatto M, Vieira L, Coelho L, Rezende D, Machado L, Mota G, Gaiato M, Santaella F, Campos E, Franco E, Callegari M, Okoshi MP, Weinreich U. Efficacy of BREATHOX® Device Inhalation on Acute Symptoms Associated with COVID-19 (BREATH Study): A Randomized Pilot Clinical Trial. J Clin Med. 2023 Sep 20;12(18):6075. doi: 10.3390/jcm12186075.

Tragoonrungsea J, Tangbumrungtham N, Nitivanichsakul T, Roongpuvapaht B, Tanjararak K. Corticosteroid nasal irrigation as early treatment of olfactory dysfunction in COVID-19: A prospective randomised controlled trial. Clin Otolaryngol. 2023 Mar;48(2):182-190. doi: 10.1111/coa.14004.

Yildiz E, Koca Yildiz S, Kuzu S, Günebakan Ç, Bucak A, Kahveci OK. Comparison of the Healing Effect of Nasal Saline Irrigation with Triamcinolone Acetonide Versus Nasal Saline Irrigation alone in COVID-19 Related Olfactory Dysfunction: A Randomized Controlled Study. Indian J Otolaryngol Head Neck Surg. 2022 Oct;74(Suppl 2):3022-3027. doi: 10.1007/s12070-021-02749-9. Epub 2021 Jul 10

Zarabanda D, Vukkadala N, Phillips KM, Qian ZJ, Mfuh KO, Hatter MJ, Lee IT, Rao VK, Hwang PH, Domb G, Patel ZM, Pinsky BA, Nayak JV. The Effect of Povidone-Iodine Nasal Spray on Nasopharyngeal SARS-CoV-2 Viral Load: A Randomized Control Trial. Laryngoscope. 2022 Nov;132(11):2089-2095. doi: 10.1002/lary.29935.

Zhang E, Zhao X, An X, Wang M, Gao J, Zhang H, Li Y. BIS-guided sedation prevents the cough reaction of patients under general anaesthesia caused by extubation: a randomized controlled trial. J Anesth Analg Crit Care. 2023 Feb 16;3(1):5. doi: 10.1186/s44158-023-00088-5.

**Table S2. In vitro effects of 0.9% saline (dilution) on/versus saliva relevant to Omicron: antibody-antigen reaction, rinse effect, infectivity (n=6 studies)**	
**A. Infectivity**	
Guang 2023	The half-life of the antigen in wet (sealed tube) samples and saline samples at room temperature was 5.0 and 2.92 days, respectively. Antigen half-life in air-dried samples at room temperature and at 4 °C was 2.93 and 11.4 days, respectively. The half-life was longer in respiratory secretions than in normal saline.
**B. Improved antibody-antigen reaction & improved (lower) detection limit**	
Kim 2022	Spike and nucleocapsid proteins as Delta and Omicron target antigens, to react with antigens of simulated gargle (human saliva + 0.9% saline): components of saliva with saline contributed to facilitating the induction of antibody-antigen binding.
Liang 2023	Use of saline enhanced Omicron detection in the saliva: saline added to 1% saliva allowed better cross-binding to Omicron antigens than 10% saliva without saline.
Zhang 2023	Better detection limit with saline (lowest threshold: 3.6×10^{-17} M and 1.6×10^{-16} M in phosphate buffered saline and untreated saliva, resp.). Pulmonary function and airway reactivity are not impacted after recovery from COVID-19 in young individuals; however, the number of symptoms reported would be associated with increased airway reactivity even after recovery in young adults who were not hospitalized with the virus.
**C. Rinse effect**	
Nguyen 2022	Study of a test device using a saline nasopharyngeal-wash: mean Ct-value was similar for saline rinse and NP swabbed sample. This supports that saline has a significant rinse effect. Also tolerability and acceptance of nasal rinse is better.
Nogueira 2022	Evaluation of rinses for contact lenses (CL): saline or several commercial rinses, used to remove virus contamination from two representative soft CL materials. Approximately 102 to 103 infectious viral particles were recovered from each CL material. Some materials were found to be more prone to coronavirus adhesion, yet contamination was already reduced to below the limit of quantification from all materials with a simple saline rinse step. Only saline rinse worked well for all materials. Other liquids were not all as reliable.
Qiao 2022	Saline gargle 0.9% (SG) versus conventional oropharyngeal swab (OPS) for Omicron detection. No significant differences between the SG and OPS results in symptomatic patients. In asymptomatic patients, the Ct -values for the SG were significantly lower than those for the OPS, implying that SG sampling had better sensitivity in the context of the Omicron variant, supporting an efficient rinse effect with a saline gargle for removing virus.

References Table 1S :

Guang Y, Hui L. Determining half-life of SARS-CoV-2 antigen in respiratory secretion. Environ Sci Pollut Res Int. 2023 Jun;30(26):69697-69702. doi: 10.1007/s11356-023-27326-1.

Jungnick S, Hobmaier B, Paravinja N, Mautner L, Hoyos M, Konrad R, Haase M, Baiker A, Eberle U, Bichler M, Treis B, Okeyo M, Streibl B, Wimmer C, Hepner S, Sprenger A, Berger C, Weise L, Dangel A, Ippisch S, Jonas W, Wildner M, Liebl B, Ackermann N, Sing A, Fingerle V; Bavarian SARS-CoV-2-Public Health Laboratory Team. Analysis of seven SARS-CoV-2 rapid antigen tests in detecting omicron (B.1.1.529) versus delta (B.1.617.2) using cell culture supernatants and clinical specimens. Infection. 2023 Feb;51(1):239-245. doi: 10.1007/s15010-022-01844-5.

Kim S, Eades C, Yoon JY. COVID-19 variants' cross-reactivity on the paper microfluidic particle counting immunoassay. Anal Bioanal Chem. 2022 Nov;414(28):7957-7965. doi: 10.1007/s00216-022-04333-8. Epub 2022 Sep 21.

Liang Y, Buchanan BC, Khanthaphixay B, Zhou A, Quirk G, Worobey M, Yoon JY. Sensitive SARS-CoV-2 salivary antibody assays for clinical saline gargle samples using smartphone-based competitive particle immunoassay platforms. Biosens Bioelectron. 2023 Jun 1;229:115221. doi: 10.1016/j.bios.2023.115221.

Nguyen TT, Zeger WG, Wadman MC, Schnaubelt AT, Barksdale AN. Pandemic driven innovation: A pilot evaluation of an alternative respiratory pathogen collection device. Am J Emerg Med. 2022 Nov;61:111-116. doi: 10.1016/j.ajem.2022.08.047.

Nogueira CL, Boegel SJ, Shukla M, Ngo W, Jones L, Aucoin MG. The impact of a rub and rinse regimen on removal of human coronaviruses from contemporary contact lens materials. Cont Lens Anterior Eye. 2022 Dec;45(6):101719. doi: 10.1016/j.clae.2022.101719.

Nguyen TT, Zeger WG, Wadman MC, Schnaubelt AT, Barksdale AN. Pandemic driven innovation: A pilot evaluation of an alternative respiratory pathogen collection device. Am J Emerg Med. 2022 Nov;61:111-116. doi: 10.1016/j.ajem.2022.08.047.

Qiao K, Tao X, Liu H, Zheng M, Asakawa T, Lu H. Verification of the efficiency of saline gargle sampling for detection of the Omicron variant of SARS-CoV-2, a pilot study. Biosci Trends. 2022 Dec 26;16(6):451-454. doi: 10.5582/bst.2022.01498.

Zhang H, Zhang C, Wang Z, Cao W, Yu M, Sun Y. Antibody- and aptamer-free SERS substrate for ultrasensitive and anti-interference detection of SARS-CoV-2 spike protein in untreated saliva. Biosens Bioelectron. 2023 Oct 1;237:115457. doi: 10.1016/j.bios.2023.115457.

To note: as viral transport media or saline, as well as saline dilution for high viral titers, have often been used for the validation of tests of Omicron, without paying attention to the sampling or dilution vehicle so far, above observations may possibly explain discrepancies in findings of the sensitivity of antibody-using tests for omicron detection (As for instance observed in Jungnick 2023).

Table S3. Effect of Nasal Saline Irrigation (SNI) or gargling in Omicron infection: proof-of-concept study in the Golden Syrian hamster: N= number of animals; TCID50=half tissue culture infective dose; dpi = days post-inoculation; [a] average comprehensive pathological scores; [b] mRNA levels of 2 critical interferon stimulated genes in lung tissues (typical of enhanced type I interferon response) in lung tissues: ISG15=interferon stimulated gene 15 and MX1=myxovirus resistance protein 1 (reflecting enhanced type I interferon response)

	**Study design**<br>**N/group & Intervention**	**Assessments**	**Parameter:**	**Nasal SI or gargling**	**Controls**	**Significance level for intergroup difference**
**SNI in Omicron infected Syrian hamsters**<br><br>**A. Efficacy study**<br><br>*(Yuan 2022)*<br><br>Omicron BA.1 variant	Experimental proof-of-concept study to assess effect on viral load (RNA and cultured viral titer) in respiratory organs and pro-inflammatory cytokines<br><br>Male Syrian hamsters intranasally inoculated with SARS-CoV-2 Omicron BA.1 variant<br><br>N=6 SNI, 1 mL, 0 to 5 days post-inoculation (dpi)<br>N=6 Controls	• Viral RNA: qPCR<br><br>• Viral titer: assessed as half tissue culture infective dose (TCID50)<br><br>• Proinflammatory cytokines in turbinate mucosa<br><br>• mRNA levels of 2 critical interferon stimulated genes in lung tissues	Survival rate:<br><br>Body weight changes:<br><br>Severity lung lesions[a]<br><br>Viral load:<br>• log10 copies/mL RNA<br>- turbinate<br>- trachea<br>- lung<br>• log10 TCID50/mL<br>• turbinate<br>• trachea<br>• lung<br><br>IL-6, IL-10, IFN-g, and TNF-a:<br><br>ISG15, MX1[b]:	100%<br><br>Rescue of body weight loss<br><br>Reduced severity<br>4.13 ± 1.69<br><br>6.27 ± 0.24<br>4.88 ± 0.65<br>5.82 ± 0.69<br><br>4.46 ± 0.58<br>2.59 ± 1.24<br>4.33 ± 0.65<br><br>Decreased (Fig 4)<br><br>Increased (Fig 4)	100%<br><br>Loss of body weight<br><br>Moderate pneumonia<br>7.67 ± 2.33<br><br>7.04 ± 0.26<br>5.91 ± 0.58,<br>7.02 ± 0.67<br><br>5.71 ± 0.37<br>4.21 ± 0.75<br>5.46 ± 0.62<br><br>(Fig 4)<br><br>(Fig 4)	 <br>*P* < 0.01<br><br>*P* < 0.0001<br><br>*P* < 0.0004<br>*P* < 0.0167<br>*P* < 0.0116<br><br>*P* < 0.0012<br>*P* < 0.0206<br>*P* < 0.0117<br><br>*P* < 0.005-0.026<br><br>*P* < 0.0018-0.0032
**B. Prophylactic study of SNI: protection against Omicron transmission**<br><br>*(Yuan 2022)*<br><br>Omicron BA.1 variant	Close-contact model of Syrian hamster infected with Omicron.<br>• N=6 donor hamsters intranasally inoculated with Omicron;<br>SNI, 1 mL daily till day 5<br>• Co-housed with N=6 recipient hamsters for 5 days<br>• Recipient hamsters euthanized at 5 dpi for virological and pathological analysis	Gross images of lung tissues of the recipient hamsters<br><br>Viral load (see Efficacy study)	Severity lung lesions in recipient hamsters[b]:<br><br>Viral load in recipient hamsters:<br>• log10 copies/mL of viral RNA<br>- turbinate<br>- trachea<br>- lung<br>• log10 TCID50/mL<br>• turbinate<br>• trachea<br>• lung	Reduced severity of lung pathology<br><br>5 to 10-fold<br>decreased (Fig 5)<br>decreased (Fig 5)<br>decreased (Fig 5)<br><br>decreased (Fig 5)<br>decreased (Fig 5)<br>decreased (Fig 5)	(Fig 5)<br>(Fig 5)<br>(Fig 5)<br><br>(Fig 5)<br>(Fig 5)<br>(Fig 5)	*P* < 0.0052<br>*P* < 0.0185<br>*P* < 0.0029<br><br>*P* < 0.0161<br>*P* < 0.0053<br>*P* < 0.0001

Table S4. Effect of Nasal Saline Irrigation (SNI) or gargling in Omicron infection: single-rinse randomised clinical trials with SNI in adults with Omicron infection :

Participants studied<br>First author [reference]<br>Study protocol & period	**Study design**<br>**N/group & Intervention**	**Assessments**	**Parameter:**	**Nasal SI or gargling**<br><br>***p-value vs BL*[a]**	**Controls/**<br>**Comparator**<br>***p-value vs BL*[a]**	**Significance level for intergroup difference**
Single gargle study<br><br>*(Bonn 2023)*<br><br>DRKS00027812<br>2022 (article referring to Omicron)	RCT, patient-blinded, single gargle study<br><br>N=30 Controls = 0.9% NaCl<br>N=31 Test (PerioAid Active; Dentaid SL) or<br>[saline = used as placebo]<br><br>Gargle, 20 mL, 60 sec	Salivary viral shedding of relevance to routine dental and otorhinolaryngological procedures<br><br>qPCR in 10 mL 0.9% NaCl gargle sample for 20 s, E-gene<br><br>Virus infectivity by culture (TCID50) at BL and 30 min after gargle	Viral load in salivary gargle sample, median:<br>• BL:<br>• 30 min:<br><br>TCID50, median:<br>• BL:<br>• 30 min:	[Saline = Placebo]<br>N=30<br>$5.1*10^5$<br>($2*10^4$; $1.4*10^7$)<br>$1.5*10^5$<br>($2.5*10^4$; $8.9*10^6$)<br>*P = 0.529*<br><br>N=9<br>6 (1, 50) PFU/mL<br>1.7 (1, 3.3) PFU/mL<br>*P = 0.0977*	[Active]<br>N=31<br>1.2×10^6<br>($8.3*10^4$; $7.5*10^6$)<br>$3.7*10^5$<br>($3.8*10^4$;$2.8*10^6$)<br>*P = 0.0435*<br><br>N=6<br>24 (7.5, 160.8) PFU/mL<br>1 (1, 1.5) PFU/mL<br>*P = 0.0313*	*Intergroup*<br>*P > 0.05*<br><br>*Intergroup:*<br>*P > 0.05*
Nasal spray efficacy study<br><br>*(Imsuwansri 2023)*<br>A. Single dose<br><br>Ancestral<br>Delta<br>Omicron BA2	RCT of nasal antibody spray or saline placebo spray (0.2 mL)<br>(randomisation 3:1):<br><br>N=9 Controls = 0.9% NaCl<br>N=27 Nasal antibody spray<br>[saline = used as placebo]<br><br>Simple nasal pump spray	SARS-CoV-2 neutralizing antibodies assessed as signal inhibition or virus neutralization potency in nasal fluid before and after placebo or nasal antibody spray application	Signal inhibition 6 hrs after single dose:<br>• Ancestral<br>• Delta<br>• Omicron BA2<br>(other mutants not reported on)	Saline placebo<br>Enhanced vs baseline<br>*P* < 0.156<br>*P* < 0.09<br>*P* < 0.062	Nasal antibody spray<br>Enhanced vs baseline<br>*P* < 0.0001<br>*P* < 0.0001<br>*P* < 0.0001	*Not mentioned*
B. Repeated-dosing tolerability study<br><br>NCT05358873	Prospective double-blind RCT assessing repeated use nasal spray 3 times/day for 2 weeks<br><br>N=9 Controls = 0.9% NaCl<br>N=27 nasal antibody spray<br>[saline = used as placebo]<br><br>Simple nasal pump spray	Range of symptoms assessed by<br>• Sino-Nasal Outcome Test-22 (SNOT-22)<br>• Self-reported Total Nasal Symptom Score (TNSS) questionnaire	% without rhinorrhoea:<br>(SNOT-22) [for other symptoms, see article]<br><br>No rhinorrhoea (TNSS)<br>No nasal congestion<br>No Nasal itch<br>No sneezing	99.2%<br>100%<br><br>100%<br>100%<br>100%<br>100%	93.4%<br>97.9%-100%<br><br>94.2<br>98.7<br>98.9<br>98.7	*P* < 0.0001<br><br>*P* = 0.0005<br>*P* > 0.9<br>*P* > 0.9<br>*P* > 0.9

BL=baseline; N=number of patients or participants; RCT=randomised clinical trial; TCID50=half tissue culture infective dose; [a] *P*-value versus baseline is given per treatment group if available.

Table S5. Clinical treatment studies: Effect of Nasal Saline Irrigation (SNI) on viral shedding and symptoms in patients with Omicron infection and tolerability: (n)RCT, quasi-experimental studies and case-control study/surveys: for Legend : see end of Table

Patient type<br>First author [reference]<br>Study protocol & period	**Baseline characteristics**	**Study design<br>N patients/group & Intervention**	**Parameters assessed**	**Results with SNI**<br><br>***p-value versus BL***[a]	**Results in controls or with comparator**<br>***p-value versus BL***[a]	**Significance level for intergroup difference**
A. Adults, not hospitalized						
**Adults, not requiring hospitalization**<br><br>*(Cegolon 2022)*<br><br>NCT05458336<br>February - March 2022:	PCR(+) asymptomatic or pre-symptomatic, or affected by mild/moderate COVID-19 symptoms<br><br>COVID-19 Antigen Rapid Test (nasopharyngeal swabs): self-test performed before nasal spray to avoid interference of spray ingredients with self-test<br><br>Vaccinated >90%	Open-label RCT: N=108<br>N=50 SOC + SNI<br>N=58 Controls (SOC)<br><br>Nasal spray: 3x/day, max. 15 days<br><br>SNI = Seawater + xylitol+ panthenol (Tonimer)[b]	VS:<br><br>NNT to achieve PCR(-) state, Day 5<br><br>Symptoms absent at study end:<br><br>OTC-medication<br>• Antipyretics<br><br>AEs	OR (CI) = 7.39 (1.83–29.8)[c]<br>HR(CI) = 6.12 (1.76–21.32)[c]<br><br>NNT=4<br><br>Most symptoms:<br>*P < 0.05-0.001*<br><br>*P = 0.323*<br><br>None treatment-related	Most symptoms:<br>*P < 0.05-0.001*<br><br>Increased: *P = 0.001*<br><br>Not reported	*P* = 0.004<br>Potential confounders: dropouts; time since onset of symptoms<br><br>*P* > 0.05<br><br>*P* > 0.05<br><br>-
Adults, presenting at Medical Laboratory Analysis sites<br><br>(de Gabory 2024)<br><br>N CT04916639<br>July 2021-March 2022	PCR (+) patients with mild/moderate COVID-19, at 15 sites, with <48 h symptoms<br><br>**<u>COVID-19</u>: 56%** (n=199)<br>• Mild: 48.3%<br>• Moderate: 51.7%<br><br>• Vaccinated: 33.5%<br><br>• Omicron: 61.3%<br>• Delta: 38.7%<br>• Alpha/wild type: 7.5%	RCT comparing:<br><br>SNI 4x/day (Physiomer spray) : N=177<br><br>**<u>COVID-19</u>**:<br><br>N=82 SNI<br>N=91 Controls<br><br>SNI, 4x/day, 3 weeks (seawater Physiomer)	**<u>COVID-19:</u>**<br>Change in Ct-value, Day5, RdRp gene (N-gene similar)<br><br>TTSR (All)<br>Severe rhinorrhoea at BL[d]<br>Severe congestion at BL[e] :<br>-Loss of smell<br>-Postnasal drip<br>-Face pain/pressure<br>-Sore throat<br>-Chest congestion<br>-Dyspnoea<br>-Headache<br>-Accomplish daily activities<br>[=~ if severe rhinorrhoea at BL]<br><br>Exacerbation to severe disease/ hospitalization	-43.6%<br><br>6.6±4.4 days<br>-2.1 days[g]<br>-1.7 days<br>3.3±1.2<br>4.5±2.7<br>3.9±1.8<br>5.9±4.5<br>3.1±1.3<br>2.9±1.5<br>4.3±4.2<br>3.7±2.7<br><br>Day7 : 9.1%<br>Day14: 0.0%<br>Day21: 0.0%	-23.9%<br><br>6.6±4.4 days<br>-<br>-<br>8.5±5.9<br>7.0±3.8<br>7.3±3.5<br>6.6±4.8<br>5.9±5.0<br>6.0±5.0<br>7.4±5.6<br>8.3±5.5<br><br>13.7%<br>12.8%<br>7.9%	*P* = 0.007<br><br>(*P < 0.05* if high load at BL)<br>*P* = 0.078<br>*P* = 0.202<br>*P* = 0.028<br>*P* = 0.037<br>*P* = 0.005<br>*P* = 0.3 (0.03[d])<br>*P* = 0.038<br>*P* = 0.019<br>*P* = 0.022<br>*P* = 0.011<br><br>NS<br>NS<br>NS

Patient type<br>First author [reference]<br>Study protocol & period	**Baseline characteristics**	**Study design<br>N patients/group &<br>Intervention**	**Parameters assessed**	**Results with SNI**<br><br>***p-value versus BL*[a]**	**Results in controls or with comparator**<br>***p-value versus BL*[a]**	**Significance level for intergroup difference**
	----------------------------------<br>**URTI:** 44%<br><br>10% with diagnosis: n=37<br>• Rhinovirus: 27.0%<br>• H enterovirus: 37.8%<br>• Influenza: 27.0%<br>• H coronavirus: 16.2%<br>• H adenovirus: 13.5%<br>• H bocavirus: 27%<br>• RSV: 10.8%<br><br>Others (n=119) : no virus/pathogen identifiable	------------------------------<br>**URTI**<br><br>All:<br>N=95 SNI<br>N=87 Controls<br><br>With aetiology:<br>N=26 SNI<br>N= 11 controls<br><br><br>SNI, 4x/day, 3 weeks (seawater Physiomer)	Household transmission:<br><br><br>----------------------------------<br>**URTI**<br>Viral load, Day3:% reduced detectability<br>Day 5: % load reduction<br><br>TTSR If no other treatment<br>All URTI:<br>- Rhinorrhea<br>- Post-nasal drip<br>- Overall sickness<br><br>If severe rhinorrhoea [g]:<br>-Postnasal drip<br>-Cough/dry cough<br><br><br>**All**<br>% relieved any aetiology:<br>Nasal congestion Day3,<br>Rhinorrhea Day3<br><br>AEs (all)<br>- nasal burning (related)<br>- serious, not SNI-related<br>1 resp. failure, 1 migraine	0-9.0%<br>If $\geq$5log10 copies/µL at BL:<br>0 – 23.8%<br>----------------------------------<br><br><br>- 62.1%<br>-25.4%<br><br>- 4.2 days<br><br>- 4.5 days<br>- 3.7 days<br>- 4.3 days<br><br><br>- 5.9 days<br>-8.4 days<br><br>-------------------------------------<br><br>89.9%<br>91.3%<br><br><br>4.3 % (8/183)<br>0.3% (1/183)<br>0.5% (2/183)	0.8 - 8.5%<br><br>0 - 36.4%<br>-----------------------------------<br><br><br>- 36.4%<br>-12.5%<br><br>-<br><br>-<br>-<br>-<br><br><br><br><br><br>-----------------------------------<br><br>71.9%<br>74.9%<br><br><br>2.8% (5/178)<br>0%<br>0%	Day 10: *P* = 0.02<br><br>Day 11 : *P* = 0.02<br>----------------------------------<br><br>-<br>*P* = 0.05<br><br>*P* = 0.045<br><br>*P = 0.037*<br>*P = 0.014*<br>*P* = 0.025<br><br>*P* = 0.037<br>*P* = 0.014<br><br>------------------------------<br><br>*P* < 0.001<br>*P* < 0.001<br><br><br>NS<br>NS<br>NS
**B. Adults in hospital during the study**						
Adults, without OGDs upon admission, kept in hospital for the study<br><br><br>*(Jing 2023)*<br><br>ChiCTR2200059651<br>5 May - 16 June 2022	PCR (+) patients from 3 hospitals admitted with COVID-19 but without OGDs on the day of admission<br><br>Assessments performed at admission and on day of discharge	DB-RCT comparing:<br><br>N=120 SNI (1x/day) + saline nasal spray + mouthwash (4x/day)<br>N=120 SNI (1x/day) + drugs (budesonide nasal spray + chlorhexidine mouthwash) (4x/day)	% (95% CI) developing OGDs<br>(Taste and Smell Survey):<br><br><br>SS (VAS)<br>- Olfactory:<br><br><br>- Gustatory:	• SNI+ spray + gargle:<br>11.8% (6.6–19.0%)<br>• SNI + drugs:<br>8.3% (4.1–14.8%)<br><br>Both interventions effective:<br>• SNI + saline: only mild<br>• SNI + drugs: 10% severe<br><br>0% moderate or severe in the saline and drug groups	40.0% (31.8–48.6%)<br><br><br>14% moderate<br>+ 19.6% severe cases<br><br>12.5% moderate<br>+ 26.8% severe cases	*P* < 0.001<br><br><br>*P* = 0 .02<br><br><br>*P* = 0.002

Patient type<br>First author [reference]<br>Study protocol & period	**Baseline characteristics**	**Study design<br>N patients/group & Intervention**	**Parameters assessed**	**Results with SNI**<br><br>***p-value versus BL***[a]	**Results in controls or with comparator**<br>***p-value versus BL***[a]	**Significance level for intergroup difference**
		N=140 Controls (no intervention)				
**Adults** with Omicron BA2.2 infection, asymptomatic or with mild or moderate COVID-19<br><br>*(Liu 2023a)*<br><br>Approved by Shandong Public Health Clinical Center<br>April - May 2022	Fever, sore throat, dry cough, hoarseness, expectoration; ≤one third symptomatic.<br><br>Moderate COVID-19 = presence of mild (X-ray) pneumonia symptoms (15% with SI, 27% controls)<br><br>Mean Ct (N gene) at study onset:<br>• 13.5 SNI<br>• 17.27 Controls (*P <0.001*)<br><br>Vaccinated: 90% Controls - 95% SNI	Quasi-experimental study: N=80<br><br>N=40 SNI Seawater 3% + SOC<br>N=40 Controls (SOC)<br><br>Spray jet system, one jet 10 sec from 10 mL per nostril + blow out nose, 2x/day until PCR(-) 2 consecutive days, or up to 21 days<br><br>SOC=Chinese (anti-flu) granules	TTSR, means:<br>• Individual symptoms<br>• Stratification by naive, refractory patients<br><br>Pneumonia cases<br><br>DVS: means, all<br>• naïve<br>• refractory<br><br>(Naive):<br>Lymphocytes:<br>Neutrophils:<br>CRP-value:<br><br>AEs not reported	Fever, sore throat, dry cough, hoarseness:<br>2 - ≤4 days<br>Expectoration:<br>8.4 days<br>(more smokers!)<br>Naive = resistant: ≤ 5 days<br>Improved after treatment<br><br>17.58 days<br>12.48 days<br>27.02 days<br><br>Increased to: 1.76.(*10^9/L)<br>Decreased: *P < 0.05*<br>Decrease to: 21.75<br><br>-	2 - ≤4 days<br><br>6.6 days<br><br>≤ 5 days<br>-<br><br>29.10 days<br>17.65 days<br>25.82 days<br><br>Unchanged: ~1.55 (*10^9/L)<br>Unchanged: *P > 0.05*<br>Slight increase to: ~28.5<br><br>-	*P* > 0.05<br><br>*P* > 0.05<br>-<br><br>*P* < 0.001<br>*P* < 0.001; *P* = 0.037 (MRA)<br>*P* = 0.888; *P* = 0.324 (MRA)<br><br>*P* < 0.05<br>*P* > 0.05<br>*P*< 0.05<br>Potential confounders: smoking, co-morbidities, BL lymphocytes
**Adults** during Omicron wave:<br><br>1. HCWs in COVID-19<br><br>(*Cao 2022)*<br><br>Communication letter<br>Omicron wave 2022	Obligatory SNI use (co-pressing measure as part of protocol strategy to reach the<br>Zero-COVID-19- strategy (Hospital cared by HCWs for 1,739 COVID-19 patients admitted to isolation wing as of February 28, 2022, and 1,836 outpatients and 832 inpatients daily in original wing 'Liu et al 2022)	Open practice survey: SNI daily prophylaxis (co-pressing measures) add-on to strict PPE<br><br>(no details on volume & frequency)<br><br>If intensive medium/high-risk occupational contact: + Molnupiravir, 5 days + Isolation 3-5 days	Response to prophylaxis: % HCWs PCR(+)<br><br>AEs not reported	Full strategy per protocol : 0%<br><br>-	(Survey HCWs: up to 84%)[f]<br><br>-	-<br><br>-
**2.** Inpatients hospitalized with COVID-19 during Omicron in a designated hospital, Shenzhen, China<br><br>*(Cao 2022)*<br>*(Liu 2022)*	Nasal SI daily as part of treatment for COVID-19<br><br>Patients present mostly with runny nose, headaches, fatigue, sneezing, and a sore throat	(R?)CT: N=140<br><br>N=68 SNI<br>N=72 Controls<br><br>SNI in the early stages of infection	DSV, survival analysis<br><br>Mortality reporting inpatients hospital (Liu 2022)<br><br>AEs not reported	Faster PCR(-) than controls<br><br>*'No deaths among inpatients'*	(see Figure)<br><br>(Survey Shanghai: 0.09%)[g]	*P* < 0.001

Patient type<br>First author [reference]<br>Study protocol & period	**Baseline characteristics**	**Study design**<br>**N patients/group &**<br>**Intervention**	**Parameters assessed**	**Results with SNI**<br><br>***p-value versus BL[a]***	**Results in controls or with comparator**<br>***p-value versus BL[a]***	**Significance level for intergroup difference**
Communication Letters<br>Omicron wave 2022	No details on SOC or other outcomes (need for ventilation/ICU)	No details on SNI strength, volume & frequency				
**Adults hospitalised with nasopharyngeal cancer under radiation therapy**<br><br>*(Yan 2024)*<br>Ethics Committee of the Fujian Cancer Hospital<br>No. K2023-207-01<br>Dec 2022 - Jan 2023	Patients requiring SNI for radiation therapy (with nasopharyngeal cancer)	Prospective (N=468):<br>Radiotherapy:<br>N=147 SNI<br>Controls:<br>N=30 Radiotherapy<br>N=291 No radiotherapy<br>N=50 HCWs<br>500 mL squeeze bottle, isotonic (37°C), 2x/day, daily maintenance	% qPCR(+)<br>% pts with fever<br><br>Peak of fever<br><br>Fever duration:<br>• Radiotherapy<br>• HCWs<br><br>AEs not reported	77.6%<br>37.%<br><br>38.32 °C<br><br><br>1.72+1.05 days	86.7-82.1%-100%<br>61.5%-54.8%<br><br>38.22 - 39.97 °C<br><br><br>2.77+2.34 days<br>3.13+1.38 days	NS<br>*P* = 0.03-0.003<br><br>NS<br><br><br>*P* = 0.008<br>*P* < 0.001
**Adults, hospitalized with pneumonia due to Omicron**<br><br>*(Pantazopoulos 2023)*<br><br><br>NCT05729204<br>June - Dec 2022	PCR(+) hospitalized patients with severe COVID-19 pneumonia, NIH category 4 (median duration of enrolment after symptom onset: 8-10 days)<br><br>Excluded: PCR(+) patients admitted for non-COVID-19-related reasons<br><br>Nasopharyngeal sampling for PCR at BL, 48 h (8 h after last wash to limit interference of ingredients with PCR-test) and Day14<br><br>Vaccinated: 54-57%	Open-label RCT:<br>N= 56<br><br>N= 28 SOC + SNI<br>N= 28 Controls (SOC)<br><br>Nasal spray: every 4 h for 16 h /day, 2 days; patients were trained in performing SNI<br><br><br>SNI = hypertonic seawater 2.3% with algal, herbal natural ingredients (Sinomarin)	VS, mean change in Ct cycles (ΔCt 48−0 h):[c]<br><br>PCR(-) Day14:<br>% (N)<br><br>HFNC or NIV:<br>% (N)<br><br>ICU admissions:<br>% (N)<br><br>Mortality Day14:<br>% (N)<br><br><br>AEs: % (N) nasal irritation	3.86 ± 3.03<br>(95%CI: 2.69 to 5.04)<br>*P < 0.001*<br><br>60.7% (17/28)<br><br><br>0% (0/28)<br><br><br>0% (0/28)<br><br><br>0% (0/28)<br><br><br>10.7% (3/28)	No change: −0.14 ± 4.29<br>(95%CI: −1.80 to −1.52)<br>*P = 0.866*<br><br>32.1% (9/28)<br><br><br>7.1% (2/28)<br><br><br>3.5% (1/28)<br><br><br>3.5% (1/28)<br><br><br>0% (0/28)	*P* < 0.001<br><br><br><br>*P* = 0.03<br><br><br>*P* > 0.05<br><br><br>*P* > 0.05<br><br><br>*P* > 0.05<br><br><br>-
**C. Adults + antiviral or antiseptic agent**						
Adults relapsing PCR(+) from 6 to 48 days after hospital discharge followed by 1 week of SNI versus no SNI after hospital discharge<br><br>*(Liao 2023)*<br><br>IRB 2022-074-02<br>15 March -30 Sept 2022	RT-PCR rebound at least one day, assessed Day7–14, 15–28, 29–45 and 46–60, after having been discharged from the hospital<br>[Higher % co-morbidities and lower Ct in re-positive versus non-re-positive patients (*P* = 0.066; <0.05)]	Retrospective matched case-control study:<br>N=3507<br>After full matching to assess rebound :<br>N=95 re-positive<br>N=129 non-re-positive<br><br>SNI: 0.9% NaCl + PVI, douche 2x/say, 5-7 days	Use of SNI if rebound after hospital discharge<br><br><br>Increase in Ct-value to normal value	Rebound(-) group: 85.3%<br><br><br>More rapid increase with SNI after Ct≥35, Day15 onwards	Rebound(+) group: 45.7%<br><br><br>Levelling off beyond Ct= 35; Ct <35 at readmission is associated with longer readmission time	*P* < 0.001<br>[No difference by vaccination status]<br><br>*P* < 0.001

Patient type<br>First author [reference]<br>Study protocol & period	**Baseline characteristics**	**Study design<br>N patients/group & Intervention**	**Parameters assessed**	**Results with SNI**<br><br>***p-value versus BL*[a]**	**Results in controls or with comparator**<br>***p-value versus BL*[a]**	**Significance level for intergroup difference**
	Vaccinated : 79-84%					
**Adults** with Omicron variant in molnupiravir study (hospitalized)<br><br>*(Zou 2022)*<br><br>ChiCTR2200056817<br>3-21 March 2022	Initial onset of symptoms for ≤5 days prior to the day of treatment<br>Patients treated in hospital<br>Mostly mild symptomatic COVID-19 (96-97%)<br><br>Median Ct for N-gene at study onset:<br>Molnupiravir/SNI: 17.98 (15.68, 21.24)<br>SNI: 17.51 (15.13, 21.43)<br><br>Median age (range):<br>Molnupiravir/SNI:39 (20,63) years<br>SNI: 42 (22, 61) years	RCT (2:1): N=107<br>N=31 basic treatment(SNI)<br>N=77 basic treatment(SNI) + Molnupiravir<br><br>Molnupiravir (800 mg twice daily, 5 days)<br><br>Daily basic treatment = SNI (volume, frequency not released) + Chinese granules<br><br>------------------------------<br>*Not discussed in article but deduced from Tables:*	TTSR, median (IQR)<br>Duration fever, median (IQR)<br><br>Primary parameter:<br>DVS median(95%CI)<br><br>% qPCR(-) Day5<br><br>% qPCR(-) Day7<br><br>% qPCR(-) Day10<br><br>--------------------------------<br>**CRP mg/mL** (7.0-7.5 at BL)<br><br>**IL-6** (higher at BL in SNI: $P < 0.029$): BL -> Treatment<br>--------------------------------<br>**AEs**	SNI: 7 (3, 7)<br>Molnupiravir/SNI 5 (3.7,7)<br>SNI: 3 (1, 3)<br>Molnupiravir/SNI 1 (1,2)<br><br>SNI: 10 (9–11)<br>+ Molnupiravir 9 (7-9)<br>SNI: 0%<br>+ Molnupiravir 18.4%<br>SNI: 6.5%<br>+ Molnupiravir 40.4%<br>SNI: 51.61%<br>+ Molnupiravir 76.3%<br>------------------------------------<br>SNI : 1.0 (0.3,6.8)<br>+Molnupravir:1.5 (0.6, 3.1)<br><br>SNI : 7.3 -> 1.6<br>+Molnupiravir: 4.6-> 1.5<br>-----------------------------------<br>SNI: 0%<br>+Molnupiravir: 3.9%<br>(ALT ↗ n=2, rash n=1)	-<br>-<br>-<br>-<br><br>-<br>-<br>-	SNI+Molnupiravir vs SNI<br>P =0.499<br><br>P = 0.096<br><br>P = 0.0092<br><br>$P < 0.001$<br><br>$P < 0.0044$<br><br>$P < 0.02$<br><br>----------------------------------<br>SNI+Molnupiravir vs SNI<br>$P > 0.05$<br><br>$P > 0.05$<br><br>-------------------------------<br>Bias? Baseline information on symptom duration prior enrolment is missing
A. Children						
**Children** with Omicron B2.2 infection, asymptomatic or mild symptomatic<br><br>*(Liu 2023b)*<br><br>GWLCZXEC2022-65<br>April-May 2022	76.6% Asymptomatic<br>Mild COVID-19<br>No moderate COVID-19 seen with Omicron.<br><br>Presenting at hospital with very low Ct values at onset of study (<20, mean ranging between 13.72-16.95); re-assessed Day 7 onwards (qPCR-detection daily)<br><br>Vaccinated 50% controls, 65% SNI	Quasi-experimental study in N=60<br><br>N=20 SNI 0.9%<br>N=20 SNI 3%<br>[Seawater + Chinese anti-flu granules]<br>N=20 Controls (Chinese granules)<br><br>Spray jet system: one jet 10 sec from 10 mL/ nostril, to blow out	TTSR, mean:<br>• Fever<br>• Cough<br><br>DVS<br><br>Lymphocyte count<br><br>% AEs:	0.9% -3% saline:<br>1.58 - 1.67 days<br>5.80 – 6.03 days<br><br>17.0 days<br><br>Increased from BL to:<br>2.25 - 2.27 (*10^9/L)<br><br><u>SNI 0.9%</u>: 0/20 (0%)<br><u>SNI 3%</u>:<br>Nasal itch: 3/20 (15%)<br>Mild pain: 2/20 (10%)	Routine treatment:<br>1.73 days<br>6.00 days<br><br>22.5 days<br><br>Unchanged<br>~1.68 (*10^9/L)<br><br>0%<br><br>0%<br>0%	 $P = 0.16$<br>$P = 0.42$<br><br>$P < 0.001$<br><br>$P < 0.05$<br><br>-<br><br>-<br>-

Patient type<br>First author [reference]<br>Study protocol & period	**Baseline characteristics**	**Study design<br>N patients/group & Intervention**	**Parameters assessed**	**Results with SNI**<br><br>***p-value versus BL[a]***	**Results in controls or with comparator**<br>***p-value versus BL[a]***	**Significance level for intergroup difference**
		nose, 2x/day until PCR(-) for 2 days				
**Children** with Omicron B2.2 infection, asymptomatic or mild symptomatic, identified during screening<br><br>*(Lin 2023)*<br><br>ChiCTR2200059802<br>April-May 2022	Outpatients<br>Runny nose, stuffy nose, cough, fever, throat hoarseness<br><br>Mild symptomatic (<40%)<br><br>Vaccinated: more controls (48.24%) than SI (33.82%) fully vaccinated (*P* < 0.003)<br><br>[Study lasting only 5 days]	Open-label RCT, cluster randomisation: N=400<br><br>N=207 SNI Physiologic seawater<br>N=200 Controls<br><br>Pump spray – 3 pumps (0.1 mL /pump) per nostril, 3x/day, for 5 days or until PCR(-) for 2 consecutive days	DVS, median<br><br>% PCR(-) Day 5<br>NNT, Day5<br>Cumulative, S-An:<br>HR (95% CI)<br>Vaccination:<br><br>TTSR (many records missing ≥Day2, not returning if PCR(-))<br><br>Hospitalization<br><br>AEs: rated as not SNI-related	2.4 days<br><br>74.88%<br>6<br>Faster to PCR(-) status<br>1.27 (1.04-1.55)<br>No interaction<br><br>SNI = Controls<br>(% with stuffy nose reduced in first 3 days)<br><br>0<br><br>Nasal pain: 1/199 (0.5%)<br>Epistaxis: 6/199 (3.0%)	3.09 days<br><br>58.78%<br><br>Slower to PCR(-) status<br>-<br>-<br><br>SNI = Controls<br><br>1/200 (0.5%)<br><br>0%<br>3/204 (1.5%)	*P* < 0.014<br><br>*P* < 0.005<br><br>*P* < 0.001 (S-An)<br>*P* = 0.017 (MRA)<br>$p_{interaction}$ = 0.363<br><br>*P* > 0.05<br>(Attrition bias)<br><br>*P* = 0.388

Abbreviations: AEs = adverse event/side effects**;** BL = baseline; Ct = threshold cycles (low = representative of high viral loads), DSS = daily symptom assessment scale; DB = double-blind; DVS = Duration viral shedding; H = human; HCWs = health care workers; HFNC = high flow nasal cannula; HS = hypertonic saline; ImS = Improvement of Symptoms; IQR = interquartile range; N = number of patients; NIV = non-invasive ventilation; NNT = number needed to treat; OGDS: = olfactory-gustatory dysfunction; PCR(+) and PCR(-) = positive and negative status following PCR-testing for SARS-CoV-2, respectively; PSR = % patients with symptom resolution; RCT = randomised clinical trial; SS = symptom severity (change); TCID50=half tissue culture infective dose; TTSR = Time to symptom resolution or to symptom relief; VAS =visual analogue scale; MRA = multiple regression analysis; S-An = survival analysis.

Explanatory notes:
[a] *P*-value versus BL is given per treatment group if available; [b] NaCl tonicity in Tonimer (called hypertonic) is unclear from composition; [c] expressed as odds ratio (OR) or hazard ratio (HR), with 95% confidence interval (95%CI), to achieve PCR(-) status vs controls; [e] Values from subgroup of COVID-19 patients with severe nasal rhinorrhoea; [e] Values from subgroup of COVID-19 patients with severe nasal congestion (for more data and *P*-values in patients with severe rhinorrhoea: see article (similar outcomes). [f] Data retrieved from Zhang et al. : the infection rate of HCWs and the reinfection of patients by the Omicron variant were obtained from Jiangsu Province, China, December 2022 to January 2023. CCDC Weekly, https://weekly.chinacdc.cn/en/article/doi/10.46234/ccdcw2023.074 ; [g] Estimation of mortality retrieved for Omicron from a survey in Shanghai: Chen X et al. Estimation of disease burden and clinical severity of COVID-19 caused by Omicron BA.2 in Shanghai, February-June 2022. Emerg Microbes Infect. 2022 Dec;11(1):2800-2807. doi: 10.1080/22221751.2022.2128435.

Table S6. Effect of Nasal Saline Irrigation (SNI) and/or gargling on COVID-19 symptoms and tolerability in Randomised Controlled trials (RCTs): Pre-Omicron studies. [Time to symptom relief; % patients with symptom resolution (PSR); Reduction in symptom severity (SS); Improvement of symptoms (ImS). Adverse events (AEs).]

	**Symptoms**	**Period**	**Study design**<br>**No. of patients/group**<br>**Intervention**	**Parameter:**	**Nasal SI or gargling**<br><br>***P-value versus BL***	**Controls/ Comparator**<br>***P-value versus BL***	**Significance level for intergroup difference**
Outpatients with COVID-19<br>*(Kimura et al.2020)*<br><br>NCT04347538<br><br>*(see also Esther et al 2022)*	Sickness, nasal congestion, cough, headache and fatigue	April 2020 - July 2020 (interim analysis)	RCT<br>(N=45; 14-17/group)<br><br>Controls<br>SNI HS (2 sachets =) 1.8%(?)<br>(SNI 1.8% + detergent)<br><br>2x/day (250 mL), 21 days	TTSR: median<br>• All symptoms<br><br>• Congestion:<br>• Headache:<br>• Cough:<br>• Fatigue:<br><br>AEs not reported	 10 days<br><br>5 days<br>3 days<br>Data missing<br>Data missing<br><br>(Called safe)	 14 days<br><br>14 days<br>12 days<br>Data missing<br>Data missing	 *P = 0.16*<br><br>*P = 0.04*<br>*P = 0.02*<br>*P = 0.19*<br>*P* = 0.17
COVID-19 patients when presenting with mild symptoms<br><br>*(Siregar 2022)* -	Fever, runny nose, nasal congestion, cough, sore throat, dysphagia, anosmia, hyposmia; no abnormal thorax X-ray	June 2020	RCT<br>(N=23/group)<br><br>Controls<br>SNI 0.9% NaCl	TTSR:<br>Mean rank time<br><br>AEs not reported	16.59 days	30.41 days	*P < 0.0001*
Asymptomatic and (81.3%) mild symptomatic COVID-19 patients<br><br>*(Chalageri 2022)*<br><br>CTR India /2020/09/027687	Cough, sore throat, malaise, loss of taste, loss of smell, aches and pains, nasal congestion; co-morbidities in 12%	Sept 2020 – Febr 2021	RCT N=80<br>(N=17-20/ group)<br><br>Controls: antipyretics, zinc, Vit C, antibiotics<br>Gargling with:<br>• HS: 20 mL 15 sec<br>• PVI: 36 mL 0.5% 30 sec<br>• Steam inhaler: 3-5 min<br><br>3x/day, 21 days	TTSR: median<br><br><br><br>Individual symptoms:<br><br><br><br>AEs not reported	All: 4.0 days<br><br><br><br>Depending on symptom<br><br><br><br>(Called safe for public)	• Controls: 7.0 days<br>• PVI : 9.0 days<br>• Steam: 5.5 days<br><br>Depending on symptom	*P < 0.01 for saline versus controls, steam & PVI*<br><br>*P < 0.01* for fever, nasal congestion, malaise<br>*P < 0.05 for* cough,<br>*P = 0.06* for sore throat
Outpatients with COVID-19 with high respiratory burden<br><br>*(Ezer 2022)*	Cough (wet or dry), shortness of breath, dyspnoea, chest congestion, or chest	Sept 2020 – June 2021 [alpha variant	RCT, DB N=203<br>(N=98 0.9% saline drops = placebo; N=105 ciclesonide inhaler/spray)	PSR (all symptoms):<br><br>PSR respiratory symptoms+fever	 Day7 : 22%<br>Day14: 45%<br><br>Day7 : 35%	Ciclesonide<br>25%<br>57%<br><br>40%	 *P > 0.05*<br><br><br>*P > 0.05*

	Symptoms	Period	Study design<br>No. of patients/group<br>Intervention	Parameter:	Nasal SI or gargling<br><br>*P-value versus BL*	Controls/<br>Comparator<br>*P-value versus BL*	Significance level for intergroup difference
NCT04435795	tightness (≤6 days)<br>Excluded:<br>• patients with only nasal or non-respiratory symptoms.<br>• vaccinated participants	March 2021]	Total daily dose:<br>- Ciclesonide: 1200 µg/mL<br>- Nasal drops 0.9% NaCl 2x/day,<br>14 days	Overall ImS:<br><br>Hospitalisation<br><br>**% with AEs:**<br>• Headache<br>• Nausea, dizziness<br>• Throat irritation<br>• Nosebleed/dry nose	Day14: 57%<br><br>Day7 : 76%<br>Day14: 93%<br><br>3/98 (3%)<br><br>15/98 (15%)<br>5/98 (5.1%)<br>2-0/98 (2%)<br>5/98 (5.1%)<br>1/98 (1%)	69%<br><br>73%<br>90%<br><br>6/105 (6%)<br><br>23/105 (22%)<br>13/105 (12%)<br>11-1/105 (12%)<br>7/105 (6.7%)<br>4/105 (3.8%)	*P > 0.05*<br><br>*P > 0.05*<br><br>*Not reported*
Symptomatic outpatients COVID-19 patients<br><br>*(Jadhav 2022)*<br><br>-	Symptom assessment only in the subpopulation, analysed after exclusion of hospitalised patients that were removed from study because of hospitalisation	Prior Octobre 2022	RCT (N=22-20/group)<br><br>SNI + gargling (0.9% NaCl)<br>up to 10x/day,<br>14 days or until feeling better	SS: mean Day 14<br>• Headache<br>• Postnasal drip<br>• Anosmia<br>• Sinusitis<br>• Sore throat<br>• Body ache<br>• Dry cough<br><br>AEs not reported	Scores 0-4<br><br><br><br><br><br><br><br><br>(Called 'safe')	Scores 1-6	 *P < 0.05*<br>*P < 0.05*<br>*P < 0.05*<br>*P < 0.05*<br>*P < 0.05*<br>*P < 0.05*<br>*P < 0.05*
Patients with non-hospitalised COVID-19:<br><br>*(Esther 2022)*<br><br>NCT04347538	WURS[a], assessing also sleep and physical activities, while various symptoms added: eye redness/pain, sputum, headache, coughing blood, shortness of breath, nausea/ vomiting, muscle/ joint pain, chills, and alteration of smell/taste	April - July 2020	RCT (N=72;N=24/group)<br><br>SNI HS (2 sachets =) 1.8%(?)<br>SNI HS 1.8%+detergent*<br>2 x/day (240 mL rinse bottles), 21 days	SS, mean nasal WURS Day 1-21[a]<br><br><br>AEs:	Scores Fig 3C[b,c]<br>Faster lowering vs. controls<br>Days 3-5-7-10-14-21<br><br>No 'safety signals':<br>No AE on smell/taste<br>No SNI-mediated spread to olfactory epithelium	Scores Fig 3C[b]	*P > 0.05*<br>after controlling for Day1 SS, RNA, other covariates[c]<br><br>[Confounders: missing BL; large variation in symptoms & their duration, summed in a cumulative WURS][c]

	Symptoms	Period	Study design<br>No. of patients/group<br>Intervention	Parameter:	Nasal SI or gargling<br><br>*P-value versus BL*	Controls/<br>Comparator<br>*P-value versus BL*	Significance level for intergroup difference
Outpatients with COVID-19<br><br>(*Soler et al. 2022)*<br><br>CONABIOS code 036-2020 in Santo Domingo, Dominican Republic, also registered as NCT04610801	Respiratory symptoms such as cough, nasal obstruction, fever, malaise without desaturation, olfactory function, anosmia<br>Excluded: hypoxia SpO2<88% to correct with oxygen, severe tachypnoea	Prior July 2022 (publication date)	RCT, DB, N=100<br><br>N=50 NS spray 0.9% NaCl<br>N=50 Xylitol + flavonoids (grapefruit seed extract) spray<br>[saline = placebo]<br><br>2 pumps/nostril, every 3 hrs, 3 days, followed by every 6 hrs, 14 days	 PSR: nasal congestion<br><br>Overall VAS<br>DSS<br>Sense of smell<br><br>Hospitalization<br><br>% AEs	 Day 4: 59.3%<br>Day 7: 82.6%<br><br>Value missing<br>Value missing<br>Value missing<br><br>0%<br><br>None reported	Xylitol<br>26.9%<br>50.0%<br><br>Value missing<br>Value missing<br>Value missing<br><br>0%<br><br>None reported	 *P* = 0.025<br>*P* = 0.017<br><br>*P* = 0.124<br>*P* = 0.448 **<br>*P* = 0.667<br><br>*P > 0.05*<br><br>-
Outpatients with COVID-19<br><br>*(Zarabanda et al. 2021)*<br><br><br>NCT04347954	Disease related symptoms<br>PCR(+) within 5 days prior enrolment<br>Fever, chills, fatigue, congestion, sore throat, smell, taste	Prior 2022	RCT, triple blind, comparator PVI<br>[saline = placebo]<br><br>N=11 NaCl 0.9%<br>N=11 PVI 0.5%<br>N=13 PVI 2.0%<br>2 x 0.1 mL nasal spray/ nostril, 4x/day,<br>5 days	SS, change vs BL:<br><br><br>ImS:<br>% patients improving<br>UPSIT:[d]<br><br>% **AEs**<br>• Nasal burning<br>• Sneezing<br>• Headache Day5<br>• Ear pain<br>• Nasal bleeds	• Fever, chills, fatigue, and congestion *P < 0.05*<br><br><br>82%<br><br>70%<br><br><br>16.7%<br>8.3%<br>16.7%<br>0%<br>0%	• PVI 0.5%: only taste: *P < 0.05*<br>• PVI 2%: all symptoms: *P < 0.05*<br><br>70-89%<br><br>73-77%<br><br><br>*28.5 - 92.9%*<br>*28.5 - 64.3%*<br>*14.3 - 42.9%*<br>*7.1 – 7.1%*<br>*7.1 – 14.3%*	*P > 0.05*<br><br>*P > 0.05*<br><br><br>*P > 0.05*<br><br>*P = 0.92*<br><br><br>*P < 0.001*<br>*P = 0.009*<br>*P = 0.22*<br>*P = 1.0*<br>*P = 0.76*

BL=baseline; DB= double-blind; RCT = randomised clinical trial; PCR(+)= positive qPCR-test. Interventions: HS=hypertonic saline; Isotonic saline=0.9% NaCl; PVI=polyvidone iodine. Outcomes: AEs=adverse event/side effects**;** DSS=daily symptom assessment scale; ImS=Improvement of symptoms; PSR= % patients with symptom resolution; TTSR=Time to symptom resolution or symptom relief; SS=symptom severity (change); VAS=visual analogue scale.

Explanatory notes: [a] Self-assessment likely directed by PCR-test to perform 4 hours after SNI; WURS = total score from an adapted Wisconsin Upper Respiratory Symptom Survey questionnaire, also integrating general well-being, non-specific and COVID-19 specific symptoms such as taste/smell disorders. [b] Results deduced from graph. [c] No Day 0 score; unclear censoring of patients; initial relief may have been missed by directing self-assessment 4 hours after rinse/SNI, while appropriateness of statistics corrected for "RNA P" can be questioned as RNA itself does not correlate with symptoms and SS in SARS-CoV-2; [d] UPSIT: University of Pennsylvania Smell Identification Test; CC = ciclesonide inhaler (600 µg 2x/ day), intranasal drops (200 µg daily).*outcome with SNI + detergent not reported; **Bias in favour of xylitol spray: study claims persistent anosmia with saline, yet this is not confirmed in Figure of smell scores showing comparable values Day28.

References Table S3:

Chalageri VH, Bhushan S, Saraswathi S, Ranganath TS, Rani VD, Majgi SM, Vijay K, Hema MS, Sanadi SL, Nasreen PM, Shoyaib KM, Partheeban I, Vanitha B, Souza ND, Vaddatti JS. Impact of Steam Inhalation, Saline Gargling, and Povidone-Iodine Gargling on Clinical Outcome of COVID-19 Patients in Bengaluru, Karnataka: A Randomized Control Trial. Indian J Community Med. 2022 Apr-Jun;47(2):207-212. doi: 10.4103/ijcm.ijcm_804_21.

Esther CR Jr, Kimura KS, Mikami Y, Edwards CE, Das SR, Freeman MH, Strickland BA, Brown HM, Wessinger BC, Gupta VC, Von Wahlde K, Sheng Q, Huang LC, Bacon DR, Kimple AJ, Ceppe AS, Kato T, Pickles RJ, Randell SH, Baric RS, Turner JH, Boucher RC. Pharmacokinetic-based failure of a detergent virucidal for severe acute respiratory syndrome-coronavirus-2 (SARS-CoV-2) nasal infections: A preclinical study and randomized controlled trial. Int Forum Allergy Rhinol. 2022 Sep;12(9):1137-1147. doi: 10.1002/alr.22975. Epub 2022 Jan 31.

Ezer N, Belga S, Daneman N, Chan A, Smith BM, Daniels SA, et al. Inhaled and intranasal ciclesonide for the treatment of covid-19 in adult outpatients: CONTAIN phase II randomised controlled trial. BMJ. 2021; 375: e068060. doi:10.1136/bmj-2021-068060.

Jadhav RB, Patil SS, Deolekar P, Yadav P. A comparative study to evaluate the use of saline nasal lavage and gargling in patients with COVID-19 infection. Int J Pram Res. 2022; 14: 12-17. EMBASE | ID: covidwho-1668051.

Kimura KS, Freeman MH, Wessinger BC, Gupta V, Sheng Q, Huang LC, et al. Interim analysis of an open-label randomized controlled trial evaluating nasal irrigations in non-hospitalized patients with coronavirus disease 2019. Int Forum Allergy Rhinol. 2020; 10(12): 1325-1328. doi: 10.1002/alr.22703.

Siregar SM, Utami RY. The Effect of Nasal Irrigation on COVID-19 Patient's Mild Symptoms of Respiratory Tract. Open Access Macedonian Journal of Medical Sciences. 2022 May 16; 10(B):1497-1501. https://doi.org/10.3889/oamjms.2022.9013.

Soler E, de Mendoza A, Cuello VI, Silva-Vetri MG, Núñez ZH, Ortega RG, Rizvi SA, Sanchez-Gonzalez M, Ferrer G. Intranasal Xylitol for the Treatment of COVID-19 in the Outpatient Setting: A Pilot Study. Cureus. 2022 Jul 23;14(7):e27182. doi: 10.7759/cureus.27182.

Zarabanda D, Vukkadala N, Phillips KM, Qian ZJ, Mfuh KO, Hatter MJ, Lee IT, Rao VK, Hwang PH, Domb G, Patel ZM, Pinsky BA, Nayak JV. The Effect of Povidone-Iodine Nasal Spray on Nasopharyngeal SARS-CoV-2 Viral Load: A Randomized Control Trial. Laryngoscope. 2022 Nov;132(11):2089-2095. doi: 10.1002/lary.29935.

Table S7. Bias assessment : studies relevant to mechanism of action (studies from Table S4)

		Randomisation process	Deviations from the intended interventions	Missing outcome data	Measurement outcome	Selection of the reported results	Overall	Comment: size of study	Comment: other
Yuan 2022	Exp	🟢	🟢	🟢	🟢	🟢	🟢	+	Experimental study
Bonn 2023	RCT	🟡	🟢	🟢	🟡	🟢	🟡	-	Saline = placebo*
Imsuwansri 2023	RCT	🟡	🟢	🟡	🟢	🟢	🟢	-	Saline = placebo

* bias against placebo by prior saline sampling for qPCR

- A red circle indicates a high risk of bias, a yellow circle indicates there are some concerns, and a green circle indicates a low risk of bias.
- Size of study: - means small study, limited number of observations; + means adequate size for its study objective; ++ means medium-sized (> 100 patients enrolled per group); [none of the studies had the large number of patients enrolled as in sponsored studies of antivirals >500]
- The qualitative bias assessment was performed, taking into account the shortcomings related to blinding of saline and its use in single-dose RCTs as placebo for the nasal spray/gargle formulations tested.
- Saline is not a drug or antiseptic, but a hygiene intervention. The assessment is done from a mechanism-of-action point of view and not from the perspective of procedural use (e.g., in dentistry).
- See main manuscript for overall problems of bias with SNI and gargling for blinding.

Table S8. Bias assessment: studies on viral shedding and symptoms in patients with Omicron infection (Studies Table S5).

		Randomisation process	Deviations from the intended intervetions	Missing outcome data	Measurement outcome	Selection of the reported results	Overall	Comment: size of study	Comment: other
Cegolon 2022	RCT	Yellow	Green	Green	Yellow	Green	Yellow	+	Role of added ingredients unclear, some concerns with randomisation and data analysis assessments
De Gabory 2024	RCT	Green	Green	Green	Green	Green	Green	++	The self-reported symptoms completed by virological assessments, reduced the possibility of biases
Jing 2024	RCT	Green	Green	Green	Green	Green	Green	++	Well performed, but patients staying in the hospital rather than at home
Liu 2023a viral load		Green	Green	Green	Green	Green	Green		Quasi experimental study, treatment-naive and refractory patients studied, effective in naive
Liu 2023a symptoms	QexS	Yellow	Green	Green	Yellow	Green	Yellow	-	Number of patients with a given symptom is very low per group,
Cao 2022 viral load reduction	nm	Yellow	Green	Green	Green	Green	Yellow	+	
Cao 2022 propylaxis	Su	Red	Green	Red	Yellow	Green	Red	++	Rather survey, saline prophylaxis as co-pressing measure;
Pantazopoulos 2023	RCT	Green	Green	Green	Green	Green	Green	+	Bias against SNI: more taste/smell dysfunctionin SNI group; standard of care not mentioned
Liao 2023	MCC	Red	Yellow	Yellow	Yellow	Yellow	Red	+	Retrospective study
Zou	RCT	Yellow	Green	Yellow	Green	Yellow	Yellow	+	SNI used as part of SOC, study randomised (2:1) for molnupiravir + SOC versus SOC
Liu 2023b viral load	QexS	Green	Green	Green	Green	Green	Yellow	+	Quasi experimentalstudy, only treatment-naive children studied; also including iso vs hypertonic saline
Liu 2023b symptoms	QexS	Yellow	Green	Green	Yellow	Green	Yellow	-	Number of patients with a given symptom is very low per group
Lin 2023 viral load	RCT	Green	Green	Green	Yellow	Green	Yellow	++	qPCR only assessed up to 5 days after randomisation
Lin 2023 symptoms	RCT	Yellow	Green	Yellow	Yellow	Green	Yellow	++	Symptomatic assessment: possible bias by selective loss-to-follow up, PCR(-) patients leaving the trial

- A red circle indicates a high risk of bias, a yellow circle indicates there are some concerns, and a green circle indicates a low risk of bias.
- Size of study: - means small study, limited number of observations; + means adequate size for its study objective; ++ means medium-sized (> 100 patients enrolled per group); [none of the studies had the large number of patients enrolled as in sponsored studies of antivirals >500]
- The qualitative bias assessment was performed, taking into account the shortcomings related to blinding of saline and its use as placebo, and evaluated as relevant in clinical practice, if causing a benefit. The blinding related performance & detection bias, which from a drug assessment perspective would be red (a high risk of bias), is not assessed from the perspective of being a drug, but of what can be achieved in clinical practice and for selfcare.
- See main manuscript for overall problems of bias with SNI and gargling for blinding.
- Bias was separately assessed for the parameter viral load and symptomatic outcomes, the summaries for both parameters found below

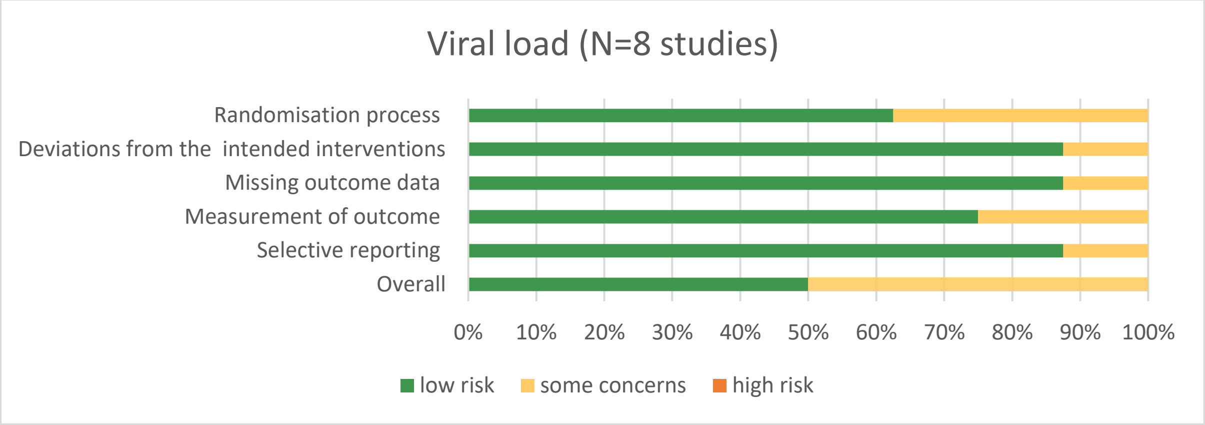
Viral load (N=8 studies)
Randomisation process
Deviations from the intended interventions
Missing outcome data
Measurement of outcome
Selective reporting
Overall
0%
10%
20%
30%
40%
50%
60%
70%
80%
90%
100%
low risk
some concerns
high risk

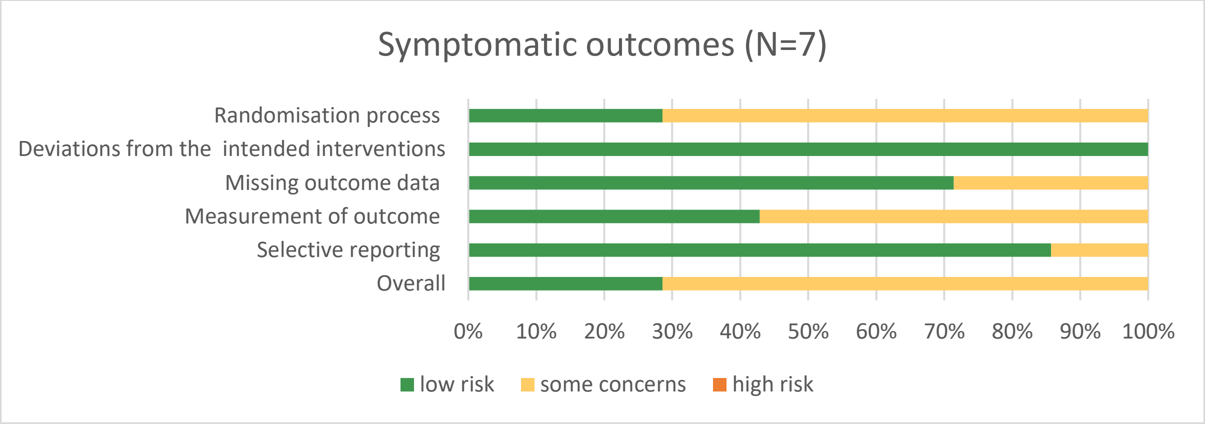
Symptomatic outcomes (N=7)
Randomisation process
Deviations from the intended interventions
Missing outcome data
Measurement of outcome
Selective reporting
Overall
0%
10%
20%
30%
40%
50%
60%
70%
80%
90%
100%
low risk
some concerns
high risk

Table S9. Bias assessment: RCTs on COVID-19 symptoms and tolerability, prior Omicron (Studies in Table S6)

Symptom assessment		Randomisation process	Deviations from the intended intervetions	Missing outcome data	Measurement outcome	Selection of the reported results	Overall	Comment: size of study	Comment : other
Kimura 2020	RCT	green	green	green	green	green	green	-	Saline = placebo
Siregar 2022	RCT	yellow	green	yellow	green	green	green	+	No info on individual symptoms
Chalageri 2022	RCT	green	green	green	green	green	green	+	Saline = placebo
Ezer 2022	RCT	green	yellow	green	green	green	green	+	Saline = placebo; stopped prematurely
Jadhav 2022	RCT	yellow	yellow	green	green	green	green	+	Hospitalised patients removed
Esther 2022	RCT	yellow	red	red	red	red	red	+	Day0 missing, controls better start values; PCRsampling 4 hrs 'after' using SNI, No info individual symptoms (only total WURS modified for COVID, incl, taste/smell disorders),
Soler 2022	RCT	green	green	yellow	green	green	green	+	Saline = placebo. Bias in data presentation in favour of xylitol
Zarabande 2022	RCT	green	green	green	green	green	green	+	Saline = placebo.

- A red circle indicates a high risk of bias, a yellow circle indicates there are some concerns, and a green circle indicates a low risk of bias.
- Size of study: - means small study, limited number of observations; + means adequate size for its study objective; ++ means medium-sized (> 100 patients enrolled per group) [none of the studies had the large number of patients enrolled as in sponsored studies of antivirals >500]
- The qualitative bias assessment was performed, taking into account the shortcomings related to blinding of saline and its use as placebo, yet as relevant in clinical practice, if causing a benefit. The blinding related performance & detection bias, which from a drug assessment perspective would be red (a high risk of bias), is not assessed from this perspective.
- See main manuscript for overall problems of bias with SNI and gargling for blinding.
- Bias summary below:

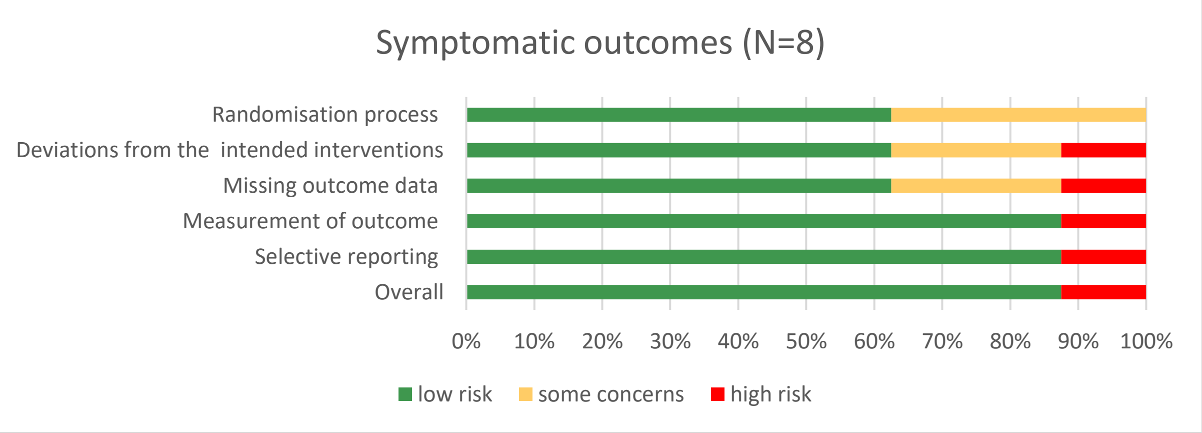

Table S10. Assessment of aggregate level of evidence:

See Yuen et al.2021 for the scores and aggregate evidence levels for non-pharmacological interventions, developed according to the 2011 Oxford Centre for Evidence Based Medicine Criteria[1]. Any disagreements amongst authors were debated per e-mail until consensus.

Aggregate evidence level	Rating	Motivation
1. Box summarizing: Viral load reduction in the nasopharynx, shorter shedding:<br>**Aggregate evidence level: B – Mainly scores 1 and 2**		
Omicron	1<br>2	RCTs (N=3): Pantazopoulos 2023: rating 1; de Gabory 2024: rating 1; Lin 2023: rating 1<br>All other (N=4) receiving rating 2 for Omicron: RCT: Cegolon 2022; quasi experimental study or undefined controlled trial design: Cao 2022, Liu 2023a, Liu 2023b
pre-Omicron (Huijghebaert 2023, Supplement)	2	• RCTs (N=6): Pantazopoulos 2022: rating 1; Chatterjee 2021; rating 1; Yilmaz: rating 2; Zarabande 2021: rating 2.<br>• Matched case control/cohort studies (N=3): Spinato 2021: rating 2; Vantakaris 2021: rating 3; Ciprandi 2021: rating 3
	2-4<br><br>3	Gargling -> salivary load:<br>• Chalageri 2022 : no effect on DVS with hypertonic saline gargling 15 mL for 15 sec, in contrast to polyvidone iodine (0.5%) 36 mL for 30 sec [bias in disfavour for saline by volume and time- period of intervention]. Yet, significant effect on symptoms with saline gargling only (not effectuated by the other mouth washes)<br>• Infectivity: consistent trend to reduced infectivity of saliva already observed 30 minutes after a single gargle 20 mL for 60 sec (pre-omicron: Gottsauner 2020; Omicron: Bonn 2023)<br>= Motivation why best to combine gargling with SNI to reduce viral load, as was also used by Pantazopoulos 2023
	[subject to bias]	Mid-turbinate load – Esther 2022: Possible biases:<br>• Bias by baseline (data not given, only Figure): Figure suggests there were already many patients in the control group with low viral loads Ct>30 on Day 1, in contrast to the irrigation groups.<br>• Low mid-turbinate load = less reliable, as can be much lower than nasopharyngeal load<br>• Self-swabbed sampling = less reliable than nurse sampled; moreover, inadequate storage of samples possible.<br>• Study is inconclusive/ tabulated data missing: there are no Day0 values; bias possible by selective leaf from the study (patients on saline evolving fast to high Ct >50 were censored (not returning? or no longer included in the outcome assessment, as seen as dots in Figure?)<br>• Potential bias by circadian rhythm effects on viral load (highest at noon/early afternoon): while controls were not imposing to wait for self-testing and for scoring symptom, sampling was delayed per protocol in the SNI groups (obligatory to wait for 4-hour after performing the SNI)<br>• Adding without validation and pooling heterogenous parameters as a total modified WURS score, moreover for analysis corrected to viral load on Day1 (while it is well known that symptoms do not correlate to viral load)

**2. *Box summarising: Symptom reduction:***<br><br>**Aggregate evidence level: B**		
	4<br><br><br><br>2<br><br><br>2	• Lack of significant symptom resolution in 4 Omicron studies (so rating 4), yet likely due to fast resolving nature, more patients on saline were not returning when PCR-negative (Ling 2023), while the sample size of symptomatic subjects was insufficient to detect statistically significant results.<br>The impact of the sample size and symptom severity is corroborated by 2 new studied 2024:<br>• de Gabory 2024: significant effect in the total randomised sample for only 2 parameters, yet on much more symptoms when analysing the subgroups with ***severe*** nasal rhinorrhoea and severe nasal congestion.<br>• Yan 2024: case-control study finding significant reduction in fever development and duration of fever, assessing fever by retrospective analysis in ***large*** cohorts
	1	• Large, well-blinded, double-dummy study of Jing 2023
pre-Omicron (Huijghebaert 2023, supplement)	2<br>3	• Five RCTs pre-Omicron: while overall well-designed, they are usually small (compared to antiviral R&D programmes)<br>• Supported by the results of matched case-control studies (Spinato 2021, Baxter 2022) evaluated in Huijghebaert 2023. Therefore, overall B
3. Box: Combination with antivirals/antiseptics:<br><br>**Aggregate evidence level C:**		
Omicron	3	Combination with antiviral: RCT with molnupiravir<br>+ based on the harm-benefit assessment of antivirals
pre-Omicron	 5<br><br><br><br><br><br>2	*This evaluation does not apply to a "single" pre-procedural use (for e.g. dentistry) or a "single (day)" post-high-risk-exposure prophylaxis.*<br>• <u>Combination of antiseptic with SNI or mouth rinse</u>, e.g. with polyvidone iodine (PVI) or chlorhexidine (CHX):<br>-> contradictory findings versus SNI/gargling without antiseptics :<br>• No benefit of combining with saline (N=2 RCTs): SNI = SNI/PVI, rating 1 (Baxter 2022), SNI = SNI/CHX rating 1 (Jing 2023)<br>• Benefit of PVI/SNI claimed over SNI: Batioglu-Karaaltin 2022: serious bias by 10x lower load in saline group at baseline; discrepancies in viral load data between text and Figure (see further Table 2S)<br>• <u>Direct comparisons with antiseptics without saline</u>: saline = antiseptic<br>• Chalageri 2022, DB-RCT, using gargling 3x/day for 21 days, finding trend to faster "nasopharyngeal" clearance with PVI (0.5%) "gargling" [6 days] versus hypertonic saline "gargling" [9 days;*P*=0.8]; yet, potential bias, as PVI gargling was performed with twice the volume and twice the rinse time of the saline gargle (time for saline gargling possibly insufficient (15 sec; usually 30-60 sec); clearance was tested in the nose rather than in the oropharynx and/or saliva. Yet symptomatic recovery was significant with SNI only (P=0.01; symptom relief seen for fever, cough, malaise, and nasal congestion with SI).<br>• Zarabande 2021, DB-RCT, finding 0.9% IS = 2.0% PVP-I > 0.5% PVP-I (4x nasal spray for 5 days): no significant reduction in viral load 1 hr after first spray application versus baseline, while decrease is significant for all 3 sprays after 3 days of application. No significant differences between treatments. Symptomatic improvement was comparable, yet a much higher % suffering adverse events with PVI (PVI 2%: 93%> 28% PVI 0.5% > SI 17%).<br>• *Procedural (single) rinse, dentistry:* not the topic of this review. Overall, unless immediately after a gargle, no significant intergroup differences in reducing viral load for saline in RCTs when compared with the antiseptic gargle (N=3: Natto 2022; Sevinç Gül 2022, Chaudhary 2021)

4. Box Summary: Prophylaxis<br><br>**Aggregate evidence level C:**		
Omicron	3	Omicron report (N=1: Cao 2023) and Case-control study (N=1: Liao 2023)
pre-Omicron (Huijghebaert 2023)	3<br>3	Harm-benefit assessment of pre-Omicron studies/reports:<br>• N=3 in Huijghebaert 2023 (see Supplement: rating 1: RCT by Gutiérrez-García et al. 2021; rating 3: Baxter 2020; rating 5: Parviz 2020)<br>• New retrospective case-control pre-Omicron study: Chuayruksa 2023b (see Table 2S)
SARS-CoV	3	Rating and aggregate level for SARS-CoV-2 studies commented in Yuen et al. Rhinology 2021 [(1)]
**5. *Box Summary: Effect of saline nasal washing on deterioration & (hospitalization) risk*:**<br><br>**Aggregate evidence level C:**		
Omicron	2<br>3<br>2<br>1	• Outcomes on inflammatory mediators (N=3: Liu 2023a, Liu 2023b, Zou 2022)<br>• Overall assessment of current Omicron studies: no patients hospitalised, while in two studies one case hospitalised among controls (de Gabory 2024, Lin 2023)<br>• Less deterioration from mild to moderate disease with SNI in study in household setting (de Gabory 2024) and less need for treatment escalation and less mortality (Pantazopoulos 2023)<br>• Yet sufficiently large RCTs are lacking.(excluding potential biases by other medication received during the hospitalization, and/or due to missing info on co-morbidities).
pre-Omicron (Huijghebaert 2023)	3<br>3	• Assessment of the risks from studies pre-Omicron (See Table S5 in Suppl to Huijghebaert 2023) – overall supporting a reduced risk, by the overall evidence presented. Yet sufficiently large RCTs (excluding potential biases by other medication received and co-morbidities) are lacking.<br>• Primary parameter in Baxter 2022, assessing two NSI regimen in a RCT, also comparing to matched controls.
Omicron + pre-Omicron	3	• New evidence: Espinoza 2023, yet no RCT but case-control study in subjects arriving at emergency depart, while no split by variant subtype (see Table S2 of this Supplement)

[1] Yuen at al 2021 used the 2011 Oxford Centre for Evidence Based Medicine Criteria. The Oxford Evidence Levels of Evidence 2. 2016 [July 4, 2020]

Table S11. PRISMA checklist:

Section and Topic	Item #	Checklist item	Location where item is reported
**TITLE**			
Title	1	Identify the report as a systematic review.	X (systematic search strategy)
**ABSTRACT**			
Abstract	2	See the PRISMA 2020 for Abstracts checklist.	x
**INTRODUCTION**			
Rationale	3	Describe the rationale for the review in the context of existing knowledge.	x
Objectives	4	Provide an explicit statement of the objective(s) or question(s) the review addresses.	X
**METHODS**			
Eligibility criteria	5	Specify the inclusion and exclusion criteria for the review and how studies were grouped for the syntheses.	X
Information sources	6	Specify all databases, registers, websites, organisations, reference lists and other sources searched or consulted to identify studies. Specify the date when each source was last searched or consulted.	X
Search strategy	7	Present the full search strategies for all databases, registers and websites, including any filters and limits used.	X
Selection process	8	Specify the methods used to decide whether a study met the inclusion criteria of the review, including how many reviewers screened each record and each report retrieved, whether they worked independently, and if applicable, details of automation tools used in the process.	X
Data collection process	9	Specify the methods used to collect data from reports, including how many reviewers collected data from each report, whether they worked independently, any processes for obtaining or confirming data from study investigators, and if applicable, details of automation tools used in the process.	X
Data items	10a	List and define all outcomes for which data were sought. Specify whether all results that were compatible with each outcome domain in each study were sought (e.g. for all measures, time points, analyses), and if not, the methods used to decide which results to collect.	X
	10b	List and define all other variables for which data were sought (e.g. participant and intervention characteristics, funding sources). Describe any assumptions made about any missing or unclear information.	X
Study risk of bias assessment	11	Specify the methods used to assess risk of bias in the included studies, including details of the tool(s) used, how many reviewers assessed each study and whether they worked independently, and if applicable, details of automation tools used in the process.	X (supplement)
Effect measures	12	Specify for each outcome the effect measure(s) (e.g. risk ratio, mean difference) used in the synthesis or presentation of results.	X
Synthesis methods	13a	Describe the processes used to decide which studies were eligible for each synthesis (e.g. tabulating the study intervention characteristics and comparing against the planned groups for each synthesis (item #5)).	X
	13b	Describe any methods required to prepare the data for presentation or synthesis, such as handling of missing summary statistics, or data conversions.	No data conversion
	13c	Describe any methods used to tabulate or visually display results of individual studies and syntheses.	X
	13d	Describe any methods used to synthesize results and provide a rationale for the choice(s). If meta-analysis was performed, describe the model(s), method(s) to identify the presence and extent of statistical heterogeneity, and software package(s) used.	Narrative from risk/benefit for selfcare, as too heterogenous study

Section and Topic	Item #	Checklist item	Location where item is reported
			designs & outcome parameters; scoring of this nonpharmacological intervention (aggregate level if evidence)
	13e	Describe any methods used to explore possible causes of heterogeneity among study results (e.g. subgroup analysis, meta-regression).	X (patient characteristics at baseline as can present diversily for selfcare, all Omicon infection, expanded to pre-Omicron in evaluation of aggregate evidence
	13f	Describe any sensitivity analyses conducted to assess robustness of the synthesized results.	No sensitivity analysis possible without (interpretative) conversions. Evaluation from risk/benefit for selfcare
Reporting bias assessment	14	Describe any methods used to assess risk of bias due to missing results in a synthesis (arising from reporting biases).	X (see supplement & discussion)
Certainty assessment	15	Describe any methods used to assess certainty (or confidence) in the body of evidence for an outcome.	X (see Method & Supplement)
**RESULTS**			
Study selection	16a	Describe the results of the search and selection process, from the number of records identified in the search to the number of studies included in the review, ideally using a flow diagram.	X
	16b	Cite studies that might appear to meet the inclusion criteria, but which were excluded, and explain why they were excluded.	X (Supplement)
Study characteristics	17	Cite each included study and present its characteristics.	X
Risk of bias in studies	18	Present assessments of risk of bias for each included study.	X (Supplement)
Results of individual studies	19	For all outcomes, present, for each study: (a) summary statistics for each group (where appropriate) and (b) an effect estimate and its precision (e.g. confidence/credible interval), ideally using structured tables or plots.	X (data as available from studies in structured tables; p-values)
Results of syntheses	20a	For each synthesis, briefly summarise the characteristics and risk of bias among contributing studies.	X
	20b	Present results of all statistical syntheses conducted. If meta-analysis was done, present for each the summary estimate and its precision (e.g. confidence/credible interval) and measures of statistical heterogeneity. If comparing groups, describe the direction of the effect.	No meta-analysis
	20c	Present results of all investigations of possible causes of heterogeneity among study results.	X
	20d	Present results of all sensitivity analyses conducted to assess the robustness of the synthesized results.	Heterogenous studies. No synthesized data, only aggregate level of evidence of existing

Section and Topic	Item #	Checklist item	Location where item is reported
			hygiene from selfcare perspective
Reporting biases	21	Present assessments of risk of bias due to missing results (arising from reporting biases) for each synthesis assessed.	(x, saline= often placebo)
Certainty of evidence	22	Present assessments of certainty (or confidence) in the body of evidence for each outcome assessed.	X
**DISCUSSION**			
Discussion	23a	Provide a general interpretation of the results in the context of other evidence.	X
	23b	Discuss any limitations of the evidence included in the review.	X
	23c	Discuss any limitations of the review processes used.	X
	23d	Discuss implications of the results for practice, policy, and future research.	X
**OTHER INFORMATION**			
Registration and protocol	24a	Provide registration information for the review, including register name and registration number, or state that the review was not registered.	Not registered (as not developed beforehand – no part of R&D programmes)
	24b	Indicate where the review protocol can be accessed, or state that a protocol was not prepared.	Not prepared, as study designs unknown ; inclusion and exclusion criteria clearly stated in Supplements
	24c	Describe and explain any amendments to information provided at registration or in the protocol.	Not applicable (for process, see Methods)
Support	25	Describe sources of financial or non-financial support for the review, and the role of the funders or sponsors in the review.	No funding, pro bono
Competing interests	26	Declare any competing interests of review authors.	No conflicts of interest
Availability of data, code and other materials	27	Report which of the following are publicly available and where they can be found: template data collection forms; data extracted from included studies; data used for all analyses; analytic code; any other materials used in the review.	Supplement

From: Page MJ, McKenzie JE, Bossuyt PM, Boutron I, Hoffmann TC, Mulrow CD, et al. The PRISMA 2020 statement: an updated guideline for reporting systematic reviews. BMJ 2021;372:n71. doi: 10.1136/bmj.n7
